# Supplementary material for: Construction of a Tm-value prediction model and molecular dynamics study of AmNA-containing gapmer antisense oligonucleotide
Source: Mol Ther Nucleic Acids. 2024 Jul 16;35(3):102272. doi: 10.1016/j.omtn.2024.102272 (PMC11339022; doi:10.1016/j.omtn.2024.102272)
Supplement: Document S1. Figures S1–S7 [file mmc1.pdf]

OMTN, Volume *35*

Supplemental information

Construction of a T_m-value prediction model and molecular dynamics study of AmNA-containing gapmer antisense oligonucleotide

Masataka Kuroda, Yuuya Kasahara, Masako Hirose, Harumi Yamaguma, Masayuki Oda, Chioko Nagao, and Kenji Mizuguchi

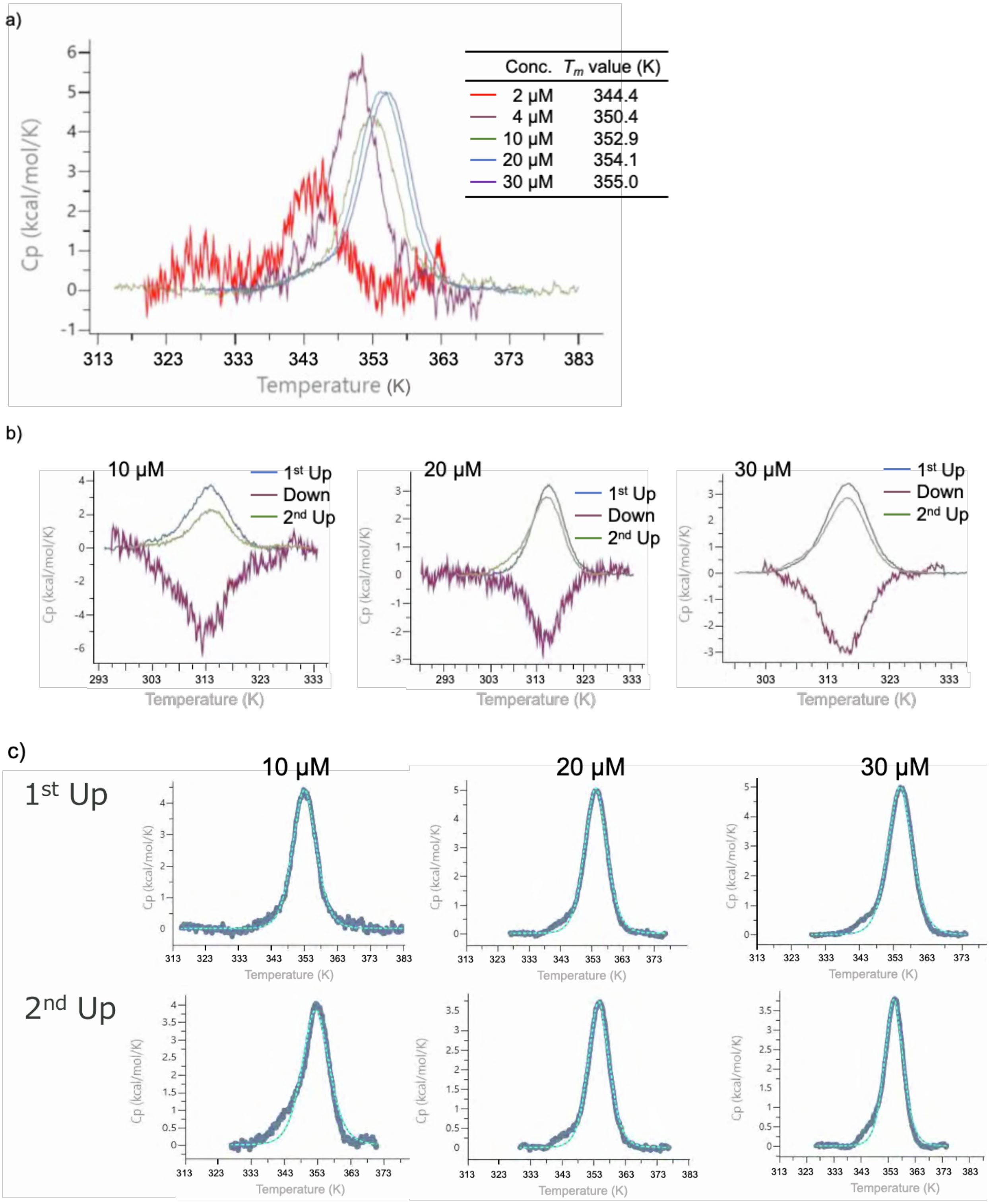

Figure S1. Differential scanning calorimetry (DSC) charts obtained from one sample.

(a) The analyzed lines of five different sample concentrations are superimposed. (b) The analysis lines for two temperature-increase and one temperature-decrease measurements are superimposed to check reversibility for three different sample concentrations. (c) The lines for two temperature-increase

measurements are superimposed to check symmetry for three different sample concentrations. The solid and broken lines indicate the measurements and the fitted line by a function, respectively.

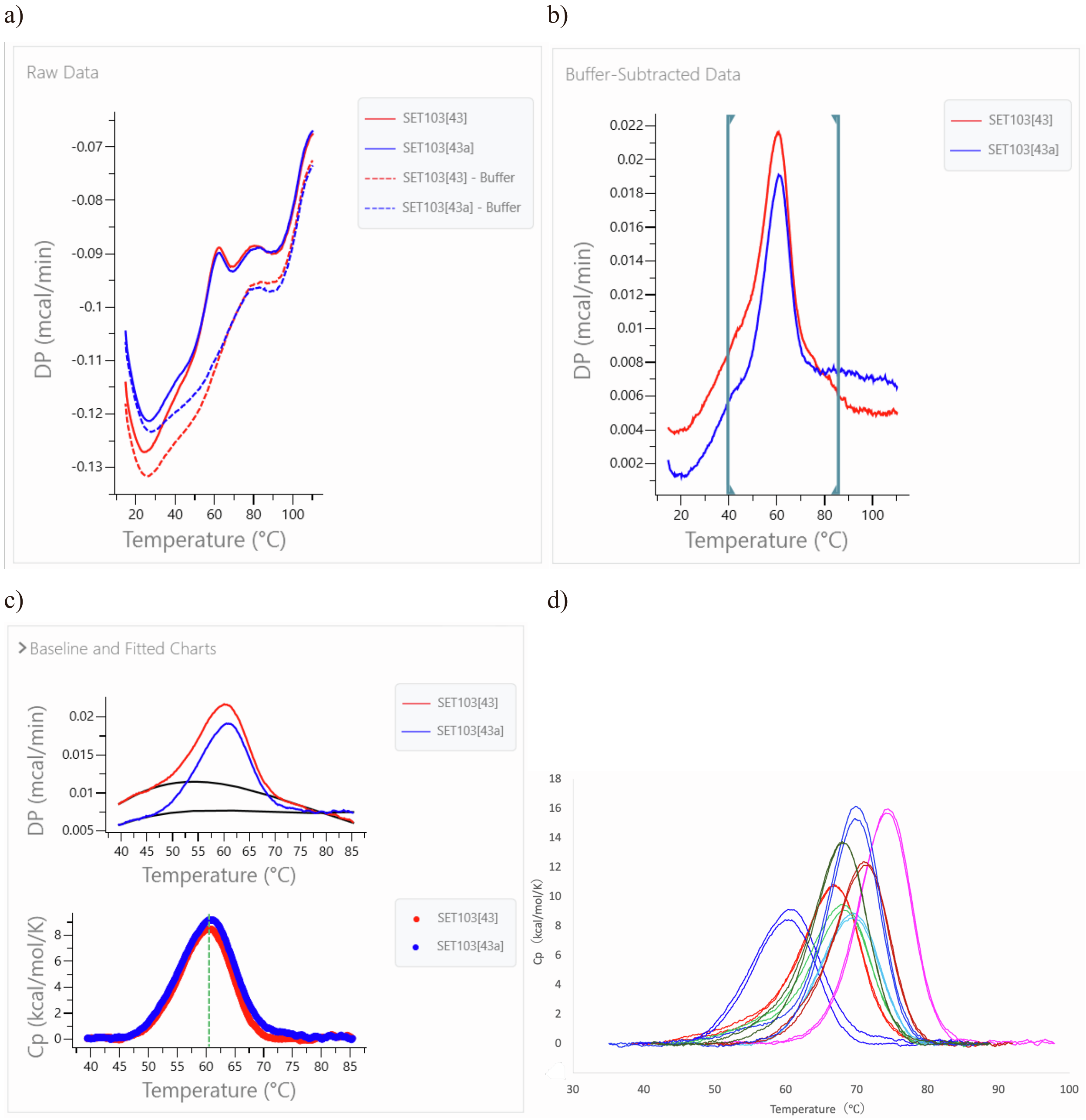

Figure S2. Measurement and Analysis Data by DSC

(a) Raw data collected for one sample. Two measurements and buffer scan data are superimposed. (b) measurement data from which the buffer data is subtracted. The area between the green bars is used for analysis. (c) Analyzed DSC data. (d) Analyzed data for eight samples are superimposed.

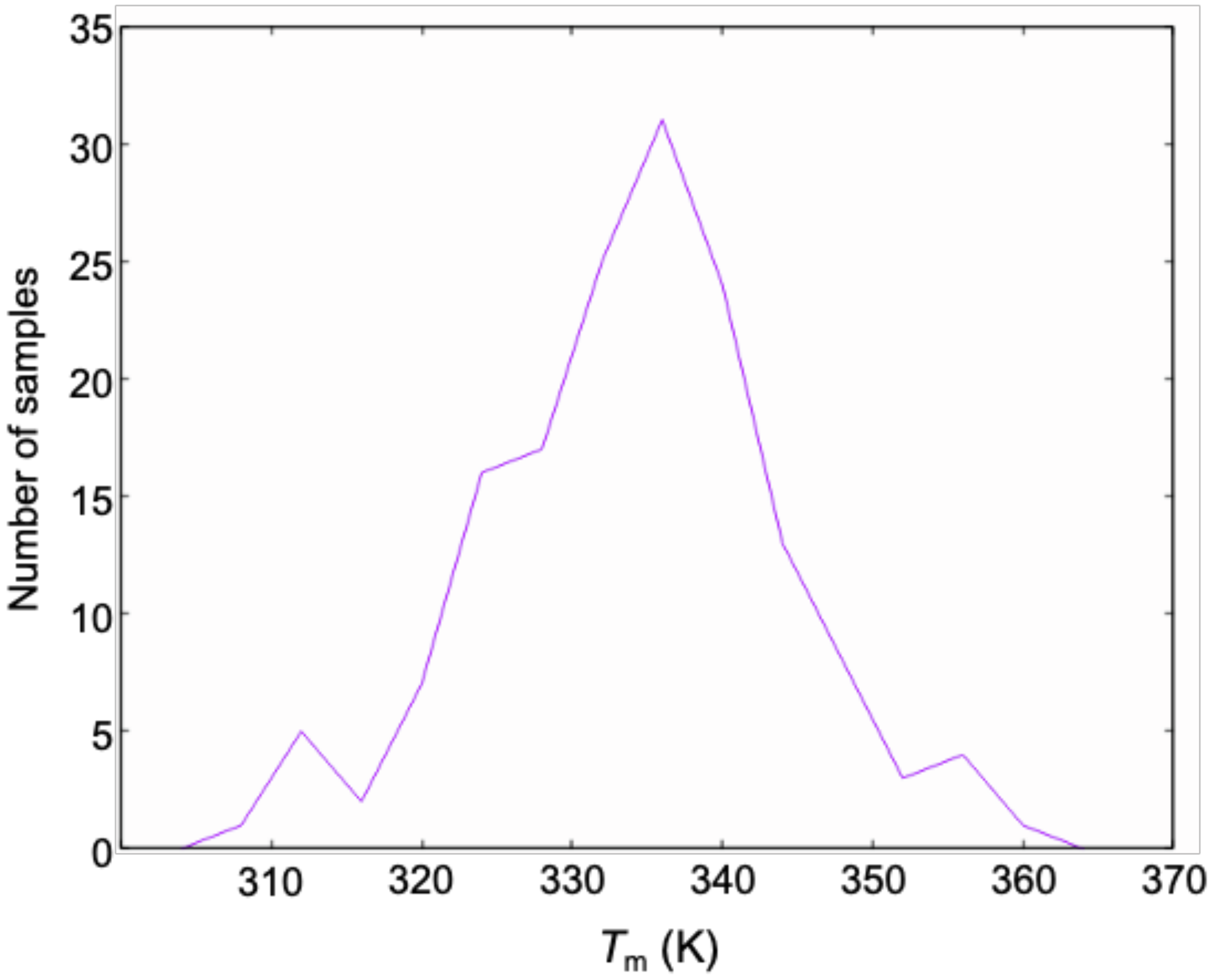

Figure S3. The Distribution of the Obtained T_m Values

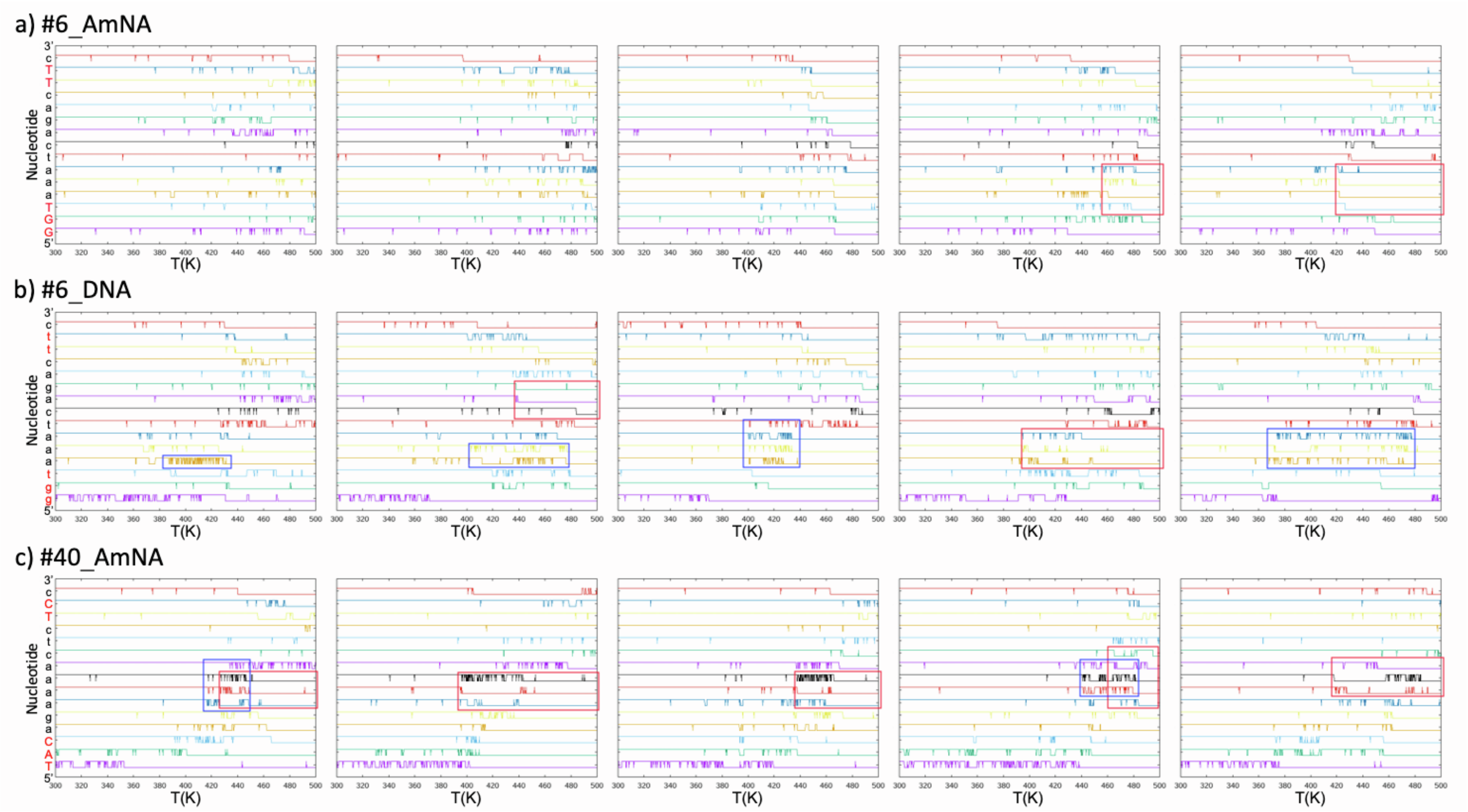
a) #6_AmNA
b) #6_DNA
c) #40_AmNA
Nucleotide
T(K)

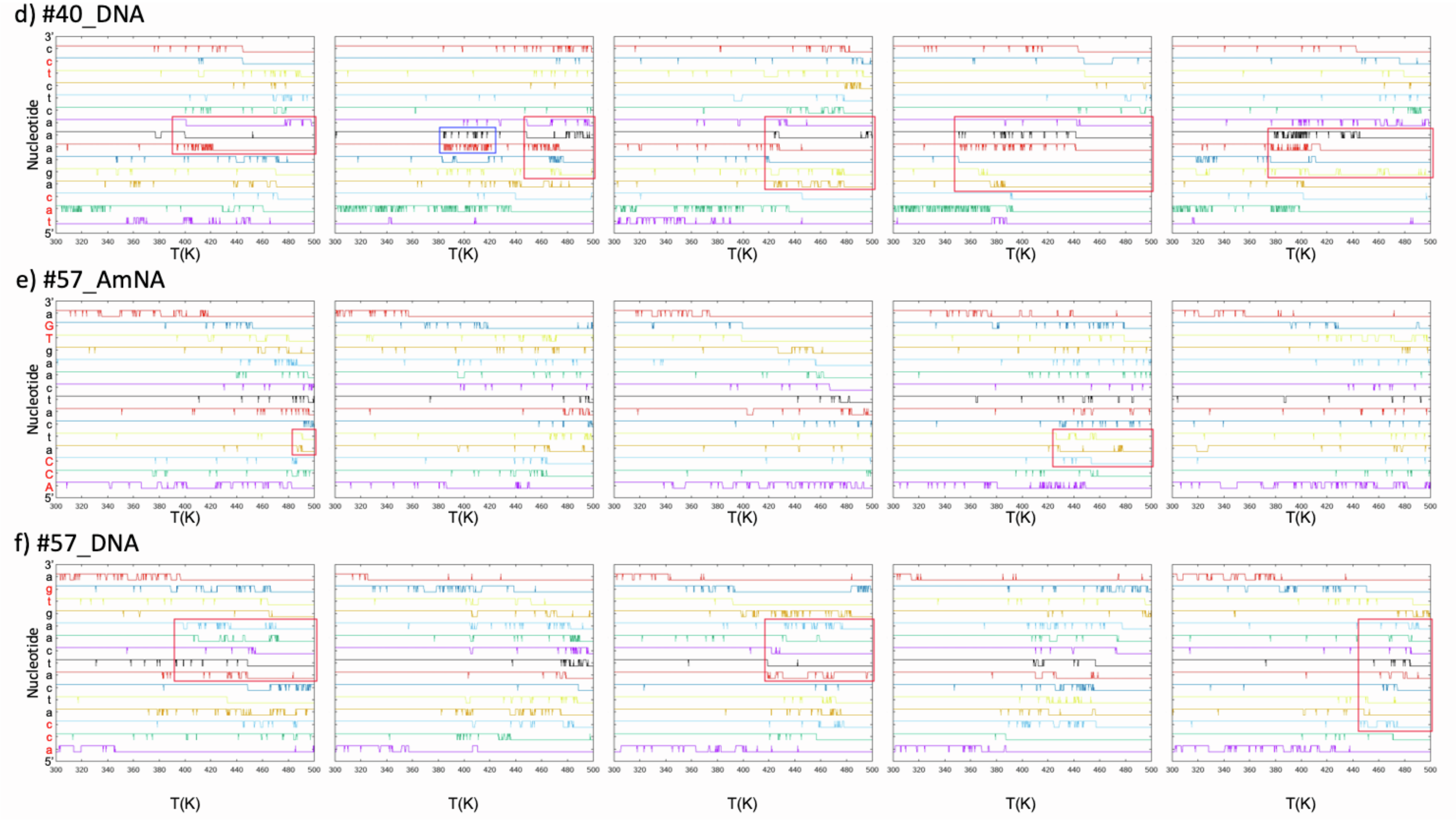

Figure S4. BPS Formation/Destruction Obtained from All MD Runs

(a) #6_AmNA, (b) #6_DNA, (c) #40_AmNA, (d) #40_DNA, (e) #57_AmNA, (f) #57_DNA. The results of run numbers one to five from the left side are displayed. The vertical axis represents the nucleotide from the 5’ to the 3’ end, with capital and small letters representing AmNA and DNA. The letter in red is the mutated position. The horizontal axis is the temperature (K) set by the MD program in the run. The top and bottom positions

of each line indicate BPS formation and non-formation, respectively. The red rectangle shows the duplex break in the middle of the oligomer. The blue rectangle shows the fray that occurs in the middle of the duplex.

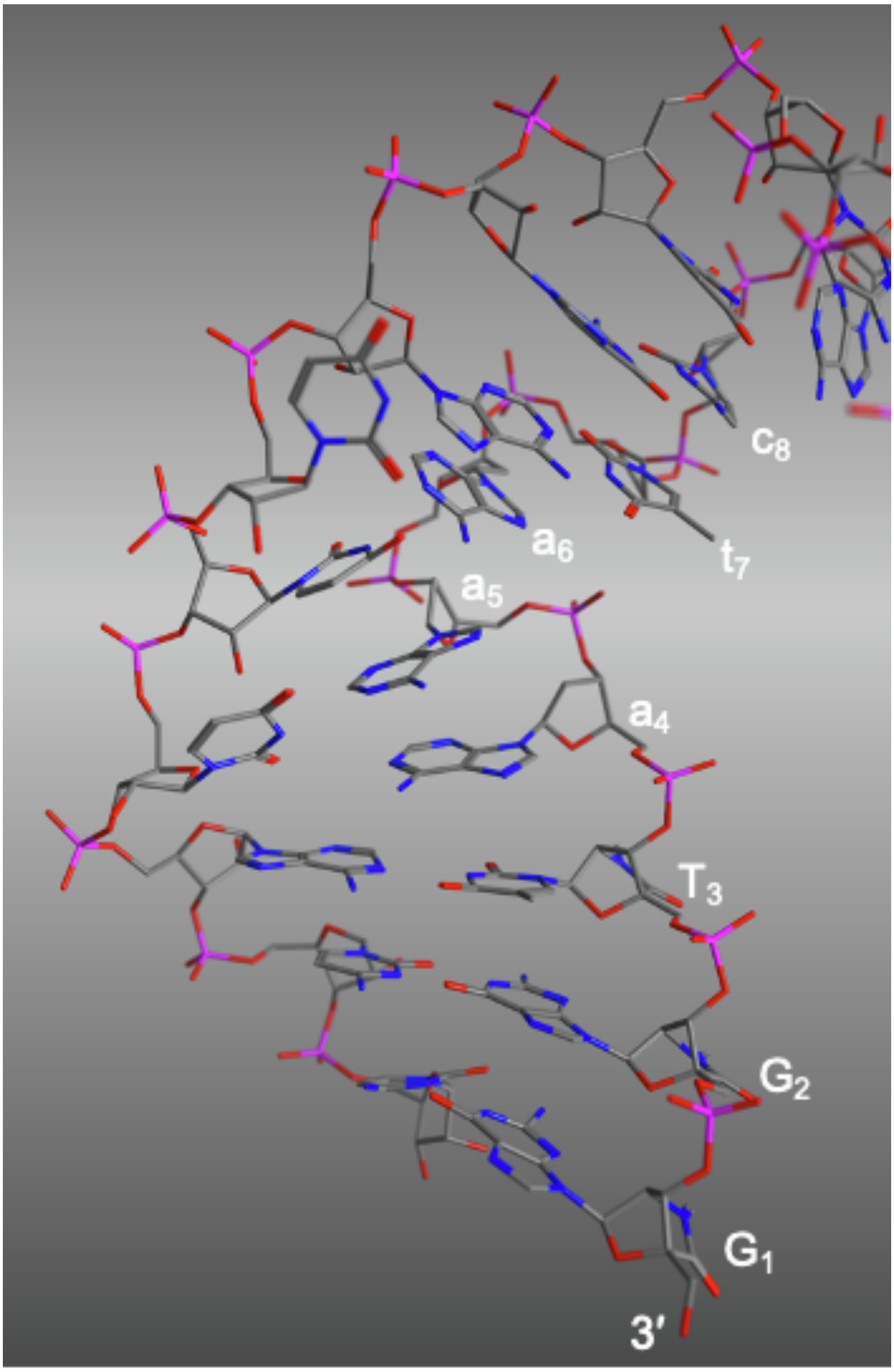

Figure S5. A snapshot of the MD trajectory for #6_AmNA 5 with the simulation temperature set to 420K, corresponding to the left side of the red rectangle in Figure 3A.

The base pair steps of the three nucleotides, a_4-a_6, are dissociated.

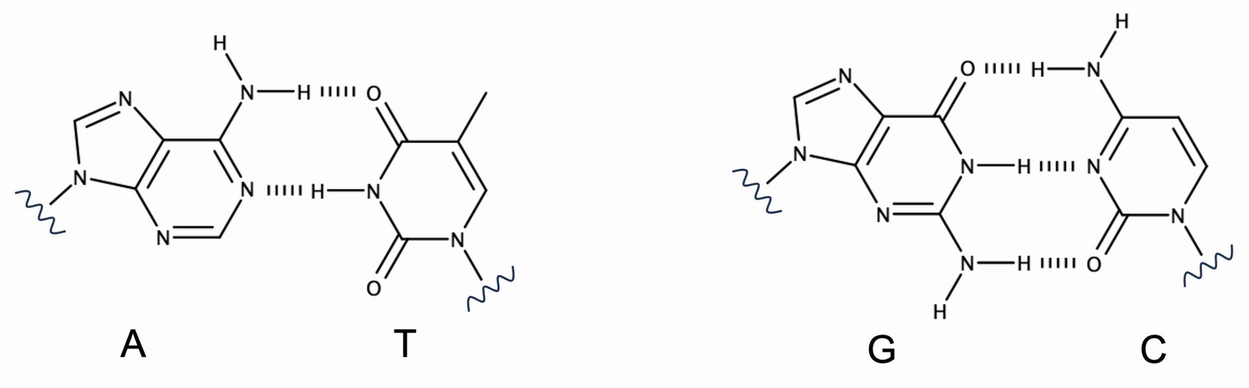

Figure S6. Interaction Modes of AT and GC Pairs

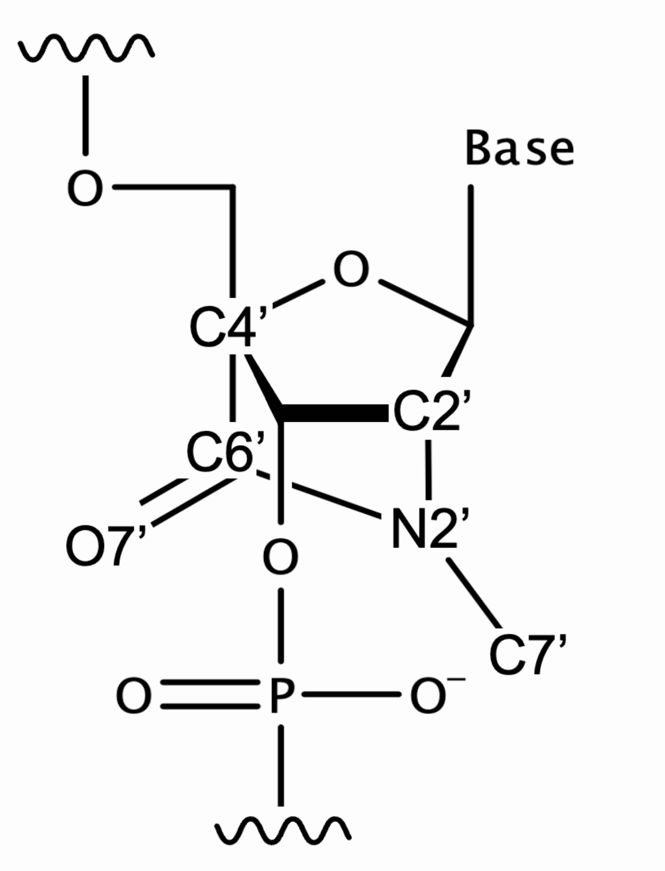

Figure S7. Atom Names of the Bridged Part in AmNA Monomer Used in Topology File for Molecular Dynamics

Table S1. T_m values for all oligonucleotides observed by DSC

Table S2. Feature importance analysis

[a]The importance values were extracted from ten models created for the performance test. They are presented as a percentage of the total importance.

Table S3. Atomic types and charges of AmNA monomers used in molecular dynamics

Atom names for the bridged part are shown in Figure S7.
