# Supplementary material for: Construction of a Tm-value prediction model and molecular dynamics study of AmNA-containing gapmer antisense oligonucleotide
Source: Mol Ther Nucleic Acids. 2024 Jul 16;35(3):102272. doi: 10.1016/j.omtn.2024.102272 (PMC11339022; doi:10.1016/j.omtn.2024.102272)
Supplement: Document S2. Article plus supplemental information [file mmc3.pdf]

Molecular Therapy
Nucleic Acids
Original Article

Construction of a T_m-value prediction model and molecular dynamics study of AmNA-containing gapmer antisense oligonucleotide

Masataka Kuroda,[1,2] Yuuya Kasahara,[1,3] Masako Hirose,[4] Harumi Yamaguma,[1] Masayuki Oda,[5] Chioko Nagao,[6] and Kenji Mizuguchi[1,6]

[1]National Institutes of Biomedical Innovation, Health and Nutrition (NIBIOHN), Osaka 566-0002, Japan; [2]Mitsubishi Tanabe Pharma Corporation, Yokohama 227-0033, Japan; [3]Graduate School of Pharmaceutical Science, Osaka University, Osaka 565-0871, Japan; [4]Malvern Panalytical, Spectris, Tokyo 105-0013, Japan; [5]Graduate School of Life and Environmental Sciences, Kyoto Prefectural University, Kyoto 606-8522, Japan; [6]Institute for Protein Research, Osaka University, Osaka 565-0871, Japan

RNase H-dependent antisense oligonucleotides (gapmer ASOs) represent a class of nucleic acid therapeutics that bind to target RNA to facilitate RNase H-mediated RNA cleavage, thereby regulating the expression of disease-associated proteins. Integrating artificial nucleic acids into gapmer ASOs enhances their therapeutic efficacy. Among these, amido-bridged nucleic acid (AmNA) stands out for its potential to confer high affinity and stability to ASOs. However, a significant challenge in the design of gapmer ASOs incorporating artificial nucleic acids, such as AmNA, is the accurate prediction of their melting temperature (T_m) values. The T_m is a critical parameter for designing effective gapmer ASOs to ensure proper functioning. However, predicting accurate T_m values for oligonucleotides containing artificial nucleic acids remains problematic. We developed a T_m prediction model using a library of AmNA-containing ASOs to address this issue. We measured the T_m values of 157 oligonucleotides through differential scanning calorimetry, enabling the construction of an accurate prediction model. Additionally, molecular dynamics simulations were used to elucidate the molecular mechanisms by which AmNA modifications elevate T_m, thereby informing the design strategies of gapmer ASOs.

INTRODUCTION

Nucleic acid medicine represents an innovative frontier, with ongoing research and development efforts for various nucleic acid therapeutics. Among these, RNase H-dependent antisense oligonucleotides (gapmer ASOs) are a class of nucleic acid drugs designed to bind target RNA, thereby recruiting RNase H for RNA cleavage to regulate the expression of proteins implicated in disease. Given that gapmer ASOs composed solely of natural nucleotides exhibit limited stability within the human body, introducing artificial nucleic acids has been a pivotal advancement in enhancing their therapeutic efficacy. Specifically, phosphorothioate and 2′-*O*-methoxyethyl (2′-MOE) nucleotides have been incorporated into commercially available drugs (Figures 1A and 1B).[1–3] Locked nucleic acid (LNA) (Figure 1C), characterized by a bridge between the 2′-*O* and 4′-*C* atoms of the ribose ring, significantly enhances the thermodynamic stability of DNA or RNA hybrid duplexes by minimizing the entropy difference upon binding.[4] LNA's utility spans numerous studies, including small interfering RNA (siRNA) and microRNA detection and therapeutic applications, demonstrating exceptional antisense potency both *in vitro* and *in vivo*.[5–8] Despite these advantages, ASOs containing LNAs face challenges, such as suboptimal pharmacokinetics and elevated hepatotoxicity.[1] Several bridged artificial nucleic acids have been developed to overcome these limitations, including amido-bridged nucleic acid (AmNA) (Figure 1D), notable for its high RNA-binding affinity and high stability due to nuclease resistance.[9,10] Additionally, it reduces the hepatotoxicity that is inherent in gapmer ASOs.[11] Consequently, we have developed various AmNA-containing gapmer ASOs to leverage their enhanced properties for therapeutic applications.[12–14]

The melting temperature (T_m) plays a critical role in the sequence design of ASOs, serving as the temperature at which equal populations of the double-stranded structure and monomer exist. Higher T_m values indicate a stronger binding affinity of the ASO to its target RNA. The nearest-neighbor method, along with its variants, is a widely recognized approach for predicting T_m values.[15–17] While studies have been predicting T_m values for oligonucleotides containing LNAs,[18–20] no specific tools exist for predicting T_m values of AmNA-containing nucleotides. As a workaround, we initially used a public T_m prediction service, substituting AmNA with LNA, which resulted in less accurate T_m predictions, often inadequate for precise sequence design. Therefore, it was difficult to use this prediction except to avoid synthesizing ASOs with T_m values that were too high or too low. For these reasons, it is required to develop a reliable T_m prediction model specific for AmNA-containing ASOs.

Received 11 April 2024; accepted 12 July 2024;
https://doi.org/10.1016/j.omtn.2024.102272.

Correspondence: Yuuya Kasahara, National Institutes of Biomedical Innovation, Health and Nutrition (NIBIOHN), Osaka 566-0002, Japan.
E-mail: y-kasahara@nibiohn.go.jp
Correspondence: Kenji Mizuguchi, Institute for Protein Research, Osaka University, Osaka 565-0871, Japan
E-mail: kenji@protein.osaka-u.ac.jp

Published by Elsevier Inc. on behalf of The American Society of Gene and Cell Therapy.
This is an open access article under the CC BY license (http://creativecommons.org/licenses/by/4.0/).

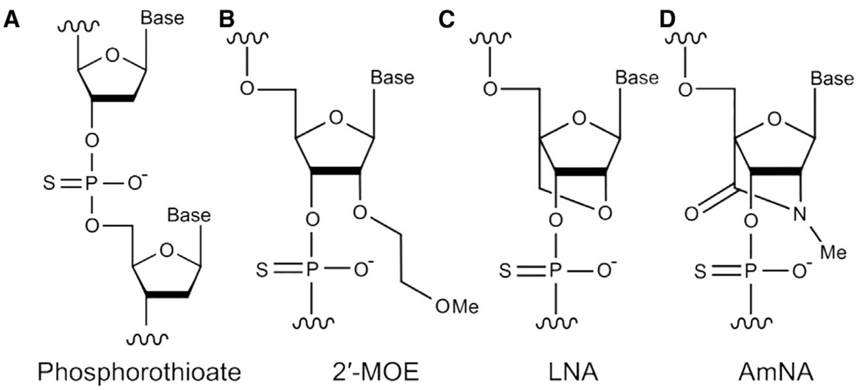

Figure 1. Chemical modifications used in nucleic acid medicine
(A) Phosphorotioate. (B) 2′-MOE. (C) LNA. (D) AmNA.

For the construction of such a model, substantial T_m measurement data would be necessary. Although the UV-visible absorption method is commonly used, it proves inefficient for analyzing many samples. Alternatively, differential scanning calorimetry (DSC) offers a more viable solution capable of detecting T_m alongside crucial thermodynamic parameters, such as enthalpy and heat capacity changes. This method facilitates the examination of molecular denaturation or unfolding by heating a solution containing the molecule.[21] The integration of an autosampler system into DSC instruments enables the automatic processing of large sample volumes once measurement conditions are established for the measurement to have been set.

In this study, we developed a highly accurate T_m prediction model by using our own library of AmNA-containing ASOs. To build this model, T_m values of more than 100 ASO oligomers were measured by DSC with an autosampler for quality control and to avoid experimental complications. In addition, we tried to clarify how mutations to AmNA increase T_m using molecular dynamics (MD) simulations.[22–24] From these analyses, a guideline for the design of gapmer ASO is also proposed.

RESULTS

T_m measurement

As a preliminary study, we identified concentration dependence, reversibility, and symmetry checks. For the concentration dependence, the T_m values tended to shift toward higher temperatures with increasing concentration (Figure S1A). The reversibility was then confirmed at each concentration (Figure S1B). Finally, the symmetry results are summarized (Figure S1C). For the first temperature-increase measurement, the symmetry tended to be worse at higher concentrations, whereas the opposite was true for the other sample. It also tended to be better at the first temperature-increase measurement than at the second temperature-increase measurement. Although the symmetry is considered to depend on the sample, the T_m values were obtained with high reproducibility.

The T_m values for 157 AmNA-containing oligonucleotide samples and three all-DNA oligonucleotide samples as references were obtained (see Table S1). Measurements for some samples are illustrated in Figure S2. The measurement lines for each sample exhibited remarkable consistency with minimal variation. While baseline distortion was observed in a few cases, the T_m values were considered reliable. The discrepancy between the two measurements for the affected samples was comparable with that observed in other samples, validating their adoption. The distribution of the T_m values obtained is depicted in Figure S3, indicating that the range of T_m values encompasses what is typically encountered in drug discovery programs.[25–27]

DSC was the method used to measure T_m values in this study. Despite the limitation of DSC in measuring only one sample at a time due to its operational mechanism, it is possible to measure up to 288 samples automatically with an auto-sampler once the measurement parameters are established. For this study, analyzing 30 samples, including dual detections per sample for quality assurance, required one night. The quantity of sample necessary for DSC, slightly exceeding that for UV-visible absorption spectroscopy—0.65 nmol (5.0 μM, 130 μL) compared with 1.7 nM (5.0 μM, 340 μL), respectively—was to ensure stable detection (refer to Figure S2) and consistency in values. Thus, the sample volume for DSC measurements is deemed acceptable. This underscores the utility of DSC in the rapid assessment of T_m values for a large volume of high-quality samples.

Prediction model

A total of 171 data points were collected. Features such as the number of neighboring nucleotide pairs and guanine or cytosine nucleobases (*nGSs*) were extracted from the sequence. The response variable was the average T_m values. As elaborated in the materials and methods section, the LightGBM framework was used to develop prediction models for the T_m values.[28]

To construct a practical prediction model for designing gapmer ASOs, the sequence diversity for the training sets was confined to nucleotide lengths between 15 and 20. The 5′-end terminus comprised three nucleotides of AmNA, while the 3′-end consisted of two AmNA nucleotides and one DNA nucleotide. Consequently, the prediction model was specifically tailored for gapmer ASOs with an approximate nucleotide length of 15.

In evaluating the model's performance, data were randomly split into training and test sets at 8:2. The mean squared error (MSE) and coefficient of determination (R^2) were computed for the test set using the prediction model derived from the training set. This process was iterated 10 times. The outcomes are summarized in Table 1, revealing an average MSE of 15.1 and an R^2 of 0.840. The MSE and

Table 1. Models' prediction performance

	MSE	R^2
New method	15.1 (5.56)	0.840 (0.0465)
Public service[a]	118. (17.8)	−0.302 (0.283)

[a]Provided by QIAGEN.[16]

R^2 obtained from the public service, calculated across all data points, were 118 and −0.302, respectively. These findings demonstrate the practical utility of the developed models for T_m value prediction in the drug research of gapmer ASOs, surpassing the performance of public services. The final model was then constructed using all data points as the training set.

MD

MD simulations were conducted to elucidate the molecular mechanisms by which mutations in AmNA elevate T_m values and to aid in design strategies for instances where similar T_m values are predicted across multiple sequences in gapmer drug discovery studies. These simulations involved incrementally increasing the temperature from 300 K to 500 K to observe the collapse of the duplex structure against six oligomers, as detailed in Table 2. Among these, three were AmNA-containing oligomers with identical GC ratios and similar T_m values, but varied AmNA substitution patterns. The other three were all-DNA oligomers, with AmNA replaced by corresponding DNA nucleotides in the former three. The phosphorothioate linkage causes stereoisomers around the phosphorus atom. It was assumed that the average duplex dissociation process of the phosphorothioate backbone would be similar to that of the phosphate backbone and the phosphate backbone was adopted in the structural models. Duplex stability was assessed by monitoring the destruction of base-pair steps (BPSs) from the simulation trajectories. Figures 2A–2C illustrate the average number of BPS formed, as derived from five MD runs for each oligomer, indicating greater stability in AmNA-containing oligomers than their all-DNA counterparts.

The subsequent stability analysis of both ends of the oligomers is presented. Figures 2D–2I depict the average number of BPSs for the three nucleotides at the 5′- and 3′-termini, respectively. At the 5′-end, replacing DNA with AmNA significantly enhanced BPS stability. Conversely, MD simulations indicated similar BPS stability at the 3′-end between AmNA-containing and all-DNA oligonucleotides across three sequences. Given that the 3′-terminal nucleotide is of DNA type for synthetic reasons, substituting AmNA at the 3′-terminus may not effectively reduce the mobility of the 3′-end. Figures 2J–2L juxtapose the BPS formation trends for the 3′-terminal nucleotide and its adjacent nucleotide (3′end-1). For oligomers where the terminal nucleobase is cytosine (**#6** and **#40**), BPS destruction at the 3′end-1 position (thymine for **#6**, cytosine for **#40**) followed closely after the 3′-end BPS destruction. In contrast, when the terminal nucleobase is adenine (**#57**), BPS destruction is initiated at a lower temperature than cytosine at the 3′-end, with

Table 2. Sequences used in MD studies

Sample ID		Sequence[a]	T_m (K)
**#6**	AmNA	GGTaaatcagacTTc	330.5
	DNA	ggtaaatcagacttc	321.1
**#40**	AmNA	TACagaaaactcTCc	330.6
	DNA	tacagaaaactctcc	321.6
**#57**	AmNA	ACCatcatcaagTGa	337.8
	DNA	accatcatcaagtga	325.9

[a]Large capital, AmNA; Small capital, DNA. All phosphate groups in all ASO oligomers are replaced with phosphorothioate. C is 5-methyl cytosine.

slower commencement at the 3′end-1. Thus, if sequences differ only by the 3′-terminal nucleobase, the T_m value for sequences with guanine or cytosine at the 3′-terminus is significantly higher than those with adenine or thymine.

The methods of BPS dissociation were further analyzed through MD simulations. Figures 3 and S4 display the formation and destruction of BPS for each nucleotide across individual MD runs, offering insight into the duplex dissociation process. Each line within these figures marks the presence (top positions) or absence (bottom positions) of BPS formation relative to temperature adjustments made by the MD software. A comparison between AmNA-containing and all-DNA oligonucleotides reveals that mutations increasing a BPS's stability typically enhance the stability of adjacent nucleotides, thereby augmenting the overall stability of the duplex. Notably, BPS disruption tends to initiate at either end of the duplex, with subsequent dissociation progressing to adjacent nucleotides. Particularly, MD simulations for specific oligomers (**#6_AmNA 4** and **5**, **#6_DNA 2** and **4**, **#40_AmNA 1**–**5**, **#40_DNA 1**–**5**, **#57_AmNA 4**, **#57_DNA 1**, **3**, and **5**) showcased BPS cleavage within the molecule's center (Figure S5), independent of the ends' cleavage, at relatively low temperatures—especially for all-DNA oligomers (as indicated by red rectangles in Figures 3 or S4). While AmNA substitution did not raise the temperature at which breaking occurred, it subtly modified the mode of breakage. For **#6_AmNA** and **#40_AmNA**, the central breakpoint shifted one or two nucleotides toward the 3′-end. In the case of **#57_AmNA**, the collapse predominantly progressed from the ends toward the center.

The relationship between sequence composition and BPS dissociation indicated that consecutive AT pairs were more prone to disruption compared with mixed AT and GC pairs, given the greater stability of GC pairs due to their three hydrogen bonds (Figure S6). Despite identical nucleotide counts and *nGCs* across the three oligomer types, **#57** exhibited a marginally higher T_m than the others. Oligomers **#6** and **#40** contained sequences with the longest runs of consecutive AT pairs ('taaat' and 'aaaa', respectively), where BPS formation proved unstable, evidenced by fraying (highlighted by blue rectangles in Figure S4 for **#6_DNA** and **#40**). Conversely, **#57** featured 'aa' or 'at' as the longest consecutive AT sequences. Although BPS disruption occurred at these sites in two runs (Figure S4 for **#57_AmNA 1**, **4**),

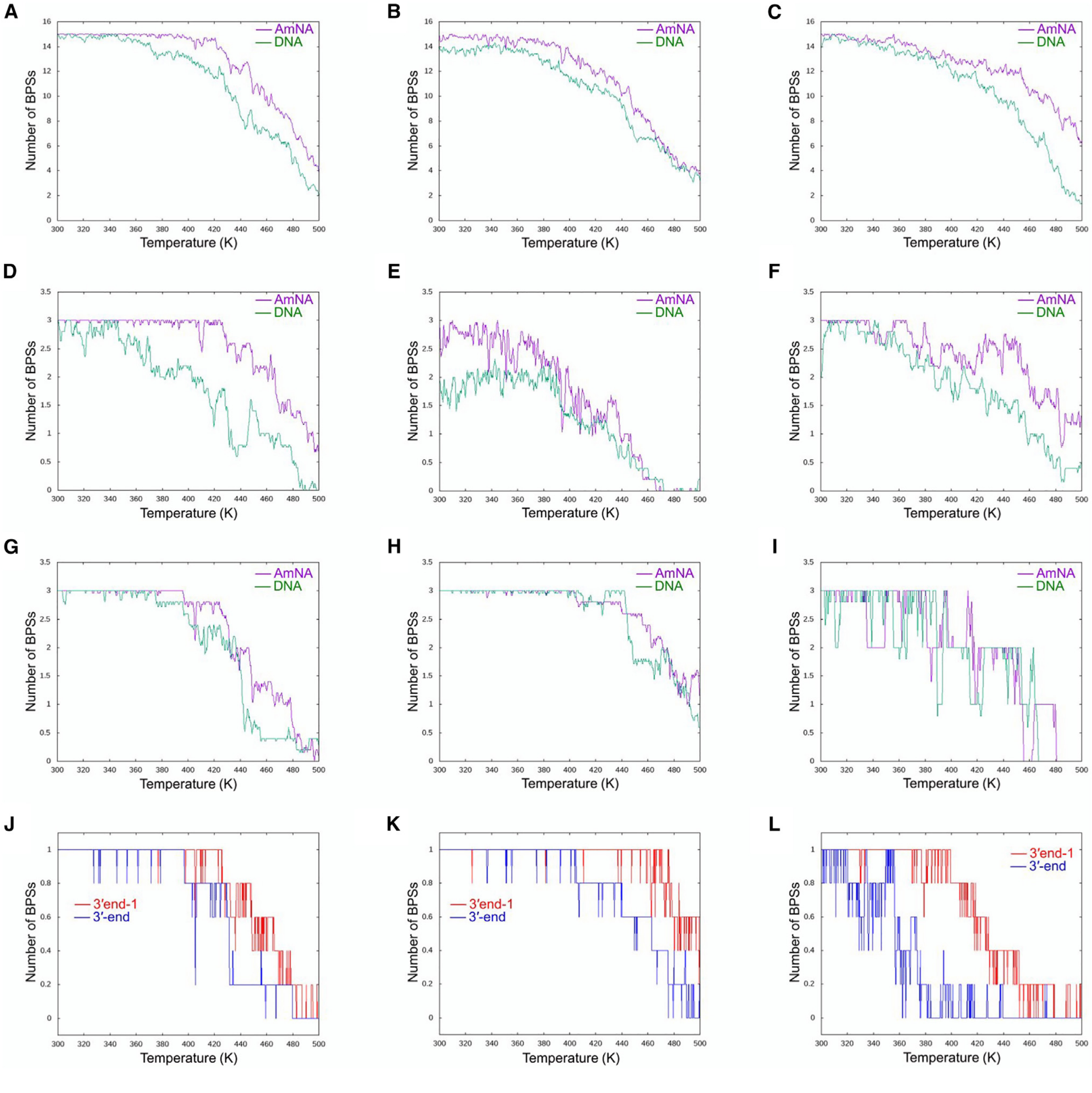

Figure 2. BPS formations obtained from five MD runs

(A–C) The average number for the whole oligomer. (D–F) The average number for three nucleotides at the 5′-end. (G–I) The average number for three nucleotides at the 3′-end. (J–L) The average number for the 3′-end nucleotide and adjacent nucleotide and the differences between the two. (A, D, G, and J) Results for **#6**. (B, E, H, and K) Results for **#40**. (C, F, I, and L) Results for **#57**. The lines for the 3′-end nucleotide and one adjacent to it are in blue and red, respectively. The vertical axis represents the number of BPSs. The horizontal axis is the temperature (K) set by the MD program in the run.

the collapse onset temperature was higher than in **#6** and **#40**. These observations were extrapolated across the entire dataset, revealing a correlation between the length of AT repetition and T_m values (illustrated in Figure 4). Thus, the arrangement of AT and GC pairs within an ASO significantly influences its T_m value.

DISCUSSION

The nearest-neighbor method, widely used for predicting the T_m values of oligonucleotides,[15–17] informed our decision to employ nucleotide pairs as predictive features. Despite the extensive AmNA-containing library, the available T_m data were limited. To

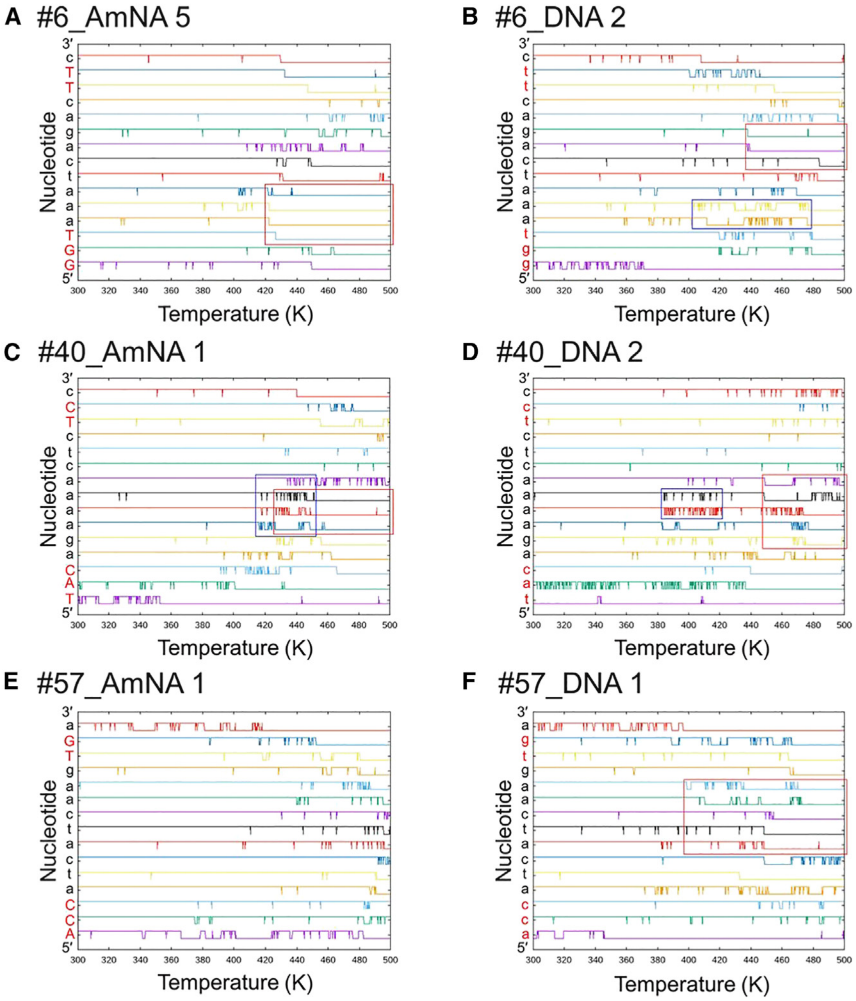

mitigate this scarcity of training data, the number of guanine or cytosine nucleobases, represented as *nGCs*, was incorporated. Feature importance was subsequently extracted from the operational model, revealing *nGCs* as the paramount predictor, accounting for 14.7% of total importance, followed by *tt* at 5.3% (refer to Table S2 for the importance of all features). A significant correlation between *nGCs* and T_m values was established, underscoring the predictive power of the *nGCs* feature (see Figure 4).

To assess the impact of this feature on prediction models, alternative models excluding *nGCs* were constructed and evaluated using performance test sets. The models yielded an average MSE of 29.3 (7.58) and an R^2 of 0.685 (0.0814), which, although inferior to prior models, surpassed the benchmarks set by public services. The exclusion of *nGCs*, defined by the count of adjacent nucleotide pairs, suggests an implicit inclusion of *nGCs*, representing the aggregate feature values in the dataset. It was observed that individual predictors within the LightGBM model cannot consider the cumulative feature count as a single feature, thereby enhancing performance when *nGCs* are explicitly included.

Figure 3. BPS formation/destruction charts obtained from MD runs

(A) #6_AmNA; Run No. 5. (B) #6_DNA; Run No. 2. (C) #40_AmNA; Run No. 1. (D) #40_DNA; Run No. 2. (E) #57_AmNA; Run No. 1. (F) #57_DNA; Run No. 1. One analysis is displayed for each sample. All results are shown in Figure S4. The vertical axis represents the nucleotide from the 5′- to the 3′-end, with capital and small letters representing AmNA and DNA, respectively. The letter in red is the mutated position. The horizontal axis is the temperature (K) set by the MD program in the run. The top and bottom positions of each line indicate BPS formation and non-formation, respectively. The red rectangle shows the duplex break in the middle of the oligomer. The blue rectangle shows the fray in the middle of the duplex.

In recent developments, the comparison of models generated by diverse machine learning techniques via user-friendly packages, such as autoML,[29–31] has gained attraction. Initially, LightGBM was used to develop baseline prediction models.[28] However, the results demonstrated sufficiently high performance for practical application in drug discovery, rendering the exploration of alternative methodologies, including random forests, support vector machines, or neural networks, unnecessary.

The conventional understanding posited that the dissociation of DNA duplexes followed a binary "all-or-nothing" model. However, specific experiments and MD simulations have suggested a more nuanced pathway involving an intermediate state characterized by terminal nucleotide fraying.[32,33] Our findings contribute to this evolving narrative by demonstrating that the dissociation process in both DNA- and AmNA-containing nucleotide duplexes is indeed more intricate. Notably, we observed fraying of the terminal nucleotide pairs in all cases, with dissociation initiating both from the periphery inward and, intriguingly, from the interior outward—the latter predominantly in AmNA-containing duplexes. This internal initiation of dissociation was not detectable in the DSC curves for the three samples, which exhibited similar profiles at the onset temperatures of dissociation. The scale of nucleotide variation in this study surpassed that of previous reports. The rapid temperature escalation in MD simulations might have skewed the accurate determination of the T_m, potentially leading to structural distortions and discrepancies with earlier findings.[32,33]

The MD simulations further elucidated that mutations at the 5′-end tend to enhance duplex stability more significantly than mutations at the 3′-end, which do not markedly increase stability. This observation is detailed in the results section. Specifically, if GC pairs recur at the 3′-end region, it's possible to reduce the number of substituted

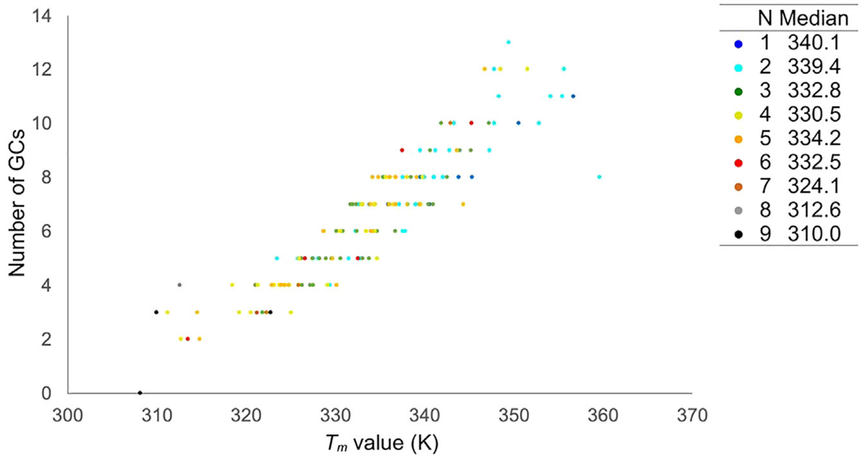

Figure 4. Correlation between the number of GCs and the T_m value

The longest number of AT repetitions in each sample is colored. The legend is listed in the table on the right, where N is the longest number of AT repetitions. The median is calculated from the values in the group of each number.

AmNAs; for instance, removing a single 3′-terminal nucleotide could maintain stability. To ascertain the impact of the 3′-terminal nucleotide, we calculated the predicted value differences between the original sequence (sequence *Org*) and its variant lacking the 3′-terminal nucleotide (sequence *3′-lack*) for the oligomers analyzed in this study. The sequences of the ASOs were classified into four groups whose two 3′-terminal nucleotides were 1; -[GC]-[at]-3′, 2; -[GC]-[gc]-3′, 3; -[AT]-[at]-3′, 4; -[AT]-[gc]-3′, where [xy] means x or y, capital and small letters represent AmNA and DNA, respectively. The 3′-terminal nucleotides of the four groups in sequence *3′-lack* are -[gc]-3′ corresponding to 1 and 2 of sequence *Org*, and -[at]-3′ corresponding to 3 and 4 of sequence *Org*. The difference is calculated by subtracting the predicted value of sequence *Org* from the predicted value of sequence *3′-lack*. The results are given in Table 3. If the nucleobase at the 3′-end is guanine or cytosine, the T_m difference is much less than that for adenine or thymine, that is, stability would be larger. Interestingly, if the 3′-terminal nucleobase is adenine or thymine and the next one is guanine or cytosine, the prediction result shows that deletion of the 3′-terminal could increase stability.

Although the production model has not been validated with external datasets, we will evaluate its performance and update it in real-life drug researches or other projects using AmNA-containing oligonucleotides. Incorporating T_m values from novel oligomers through active learning[34] or similar methodologies underscores the efficacy of our predictive model, constructed from merely 157 data points across various oligomer types, including those containing artificial nucleic acids. It was also shown that the method to make predictive models would be applied to oligonucleotides including other types of artificial nucleotides if data on T_m values are collected.

The model created in this study cannot be used to predict clinical efficacy or safety profile because the model only gives the T_m value of the ASO sequence. However, in the early stage of a new ASO drug discovery program, T_m prediction can help to avoid inappropriate sequences other than those with high GC content. In this stage, the number of samples is expected to be at most halved compared with conventional methods.

In summary, the study established a highly accurate T_m prediction model based on DSC observations and MD insights, highlighting two critical aspects for gapmer ASO design: the thermodynamic stability depends on the nucleobase combination at the 3′-end, particularly favoring guanine or cytosine, and the repetition of adenine or thymine diminishes duplex stability. While directly correlating T_m value to ASO activity is challenging due to the influence of mRNA's three-dimensional conformation on ASO-mRNA association, this research offers valuable tools and insights for understanding ASO thermodynamic stability.

MATERIALS AND METHODS

Sample preparation

A collection of 157 oligonucleotides was prepared, comprising 127 selected from our laboratory's gapmer repository and 30 that were newly conceptualized. The criteria for generating new sequences included a GC content ratio below 0.8 and distinct sequence homology, ensuring minimal similarity to both previously created sequences and those within the existing pool. These oligonucleotides were acquired from Ajinomoto Bio-Pharma Services (Osaka, Japan). For synthetic purposes, each monomer of all gapmer ASOs was modified with a phosphorothioate group to enhance stability by nuclease resistance. Additionally, synthetic RNA oligomers were procured from Thermo Fisher Scientific K.K. (Tokyo, Japan) to complement the 157 gapmers to facilitate the study. All oligomers were of HPLC grade.

T_m observation

MicroCal PEAQ-DSC automated (Malvern Panalytical, Northampton, MA, USA) was used to obtain the T_m values of ASOs. All ASOs and complementary RNAs were prepared to a respective final concentration of 5 μM in 1 × PBS buffer. Thermal scans were conducted within the temperature range of 283 K–338 K at a 120 K/h scanning rate. Data was analyzed using the PEAQ-DSC Analysis software from Malvern Panalytical. The T_m value for each ASO was established as the mean of two separate measurements to ensure accuracy and reproducibility.

Construction of T_m prediction models

Leveraging the widely adopted nearest-neighbor method[15–17] for T_m prediction, the adjacent nucleotide pair was designated a feature for machine learning analysis. Given the distinct environments of the

Table 3. Effect of the deletion of 3′end sequence on T_m predictions

Sequence *Org*[a]	Sequence *3′-lack*[b]	Averaged $\Delta T_m^{pred.}$[c]
-[GC]-[at]-3′	-[gc]-3′	1.09
-[GC]-[gc]-3′	-[gc]-3′	−2.55
-[AT]-[at]-3′	-[at]-3′	−0.447
-[AT]-[gc]-3′	-[at]-3′	−3.01

[a]Sequences used for T_m observation.
[b]Sequences in which the 3′-terminal nucleotide of sequence *Org* is deleted.
[c]The predicted value for sequence *Org* was subtracted from the predicted value for sequence *3′-lack*.

5′- and 3′-terminal nucleotides, compared with those nestled between adjacent nucleotides, pair features incorporating terminal nucleotides were separately identified. The feature count totaled 192, reflecting the presence of eight nucleotides and three types of nucleotide pair configurations: 5′-middle, middle-middle, and middle-3′, as depicted in Figure 5A. In this research, AmNA modifications were consistently applied at positions 1, 2, 3, −3, and −2, with DNA monomers exclusively positioned at the 3′-end, leading to an equal distribution of four nucleotides at both the 5′- and 3′-ends, thereby reducing the feature count to 128. The number of each pair was set to the feature. Figure 5B shows how to set values for the given sequence, CgagaGc, where capital and small letters represent AmNA and DNA, respectively. The number of guanine or cytosine nucleobases (*nGCs*) was added to the features. Feature selection within the training set was executed as follows: a feature was eliminated if the correlation coefficient between any two features exceeded 0.9; a feature was removed if the prevalence of the most frequent value over the second-most frequent was greater than 19.0 or if the ratio of distinct values to the total sample count was less than 0.1.

The LightGBM framework was used for model development.[28] Data were divided into training and validation sets at a 9:1 ratio. The hyperparameters set included n_estimators at 1,000, nfold at 5, and early_stopping_rounds at 100. Further parameter optimization was achieved through Optuna,[35] encompassing a range of values for learning_rate; [0.001, 0.1], max_depth; [1, 100], max_bin; [255, 500], lambda_l1; [1e−8, 10.0], lambda_l2; [1e−8, 10.0], num_leaves; [2, 256], feature_fraction; [0.4, 1.0], bagging_fraction; [0.4, 1.0], bagging_freq; [1, 7], min_child_samples; [5, 100], where values in brackets indicate the explored minimum and maximum bounds. The production model was built using all samples as the training set.

Performance test

All samples were divided into training and test sets following an 8:2 ratio. A model was then created using the training set, with the MSE and R^2 calculated based on the test set. This process was repeated ten times independently to ensure reliability. The performance of our prediction models was benchmarked against the public prediction service offered by Qiagen,[18] under specified experimental conditions for RNA; 1 μM, Na^+; 115 mM, Mg^{2+}; 0 mM.

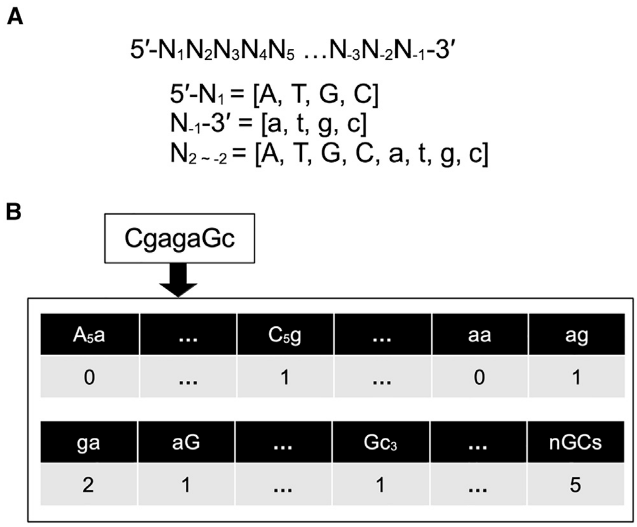

Figure 5. Features used in machine learning
(A) Nucleotides in the oligonucleotides used in this study. N_i, nucleotide at position i. Capital and small letters represent AmNA and DNA, respectively. (B) Representation of features for a sample sequence. Features are 128 adjacent nucleotide pairs and number of GC pairs (*nGCs*). Each value represents the frequency of the feature.

MD

Three sequences were selected, as shown in Table 2, in which the GC ratios were identical, the T_m values were similar, and the AmNA substitution patterns were varied. The duplex models were initially generated using RNA monomers in the A form, substituting RNA monomers corresponding with DNA in the ASO with DNA and manually adjusting the sugar rings at AmNA positions. The modeling process involved two stages of energy optimization: first, minimizing the energy of the sugar ring and added atoms while fixing other atoms' coordinates, followed by minimizing the entire conformation's energy using Molecular Operating Environment (MOE).[36]

The molecular complexes were immersed in TIP3P water within an 80 Å cube. Sodium ions were added to neutralize the charge, and both sodium and chloride ions were introduced to achieve a 100-mM concentration in each system, using the AMBER94 force field for DNA monomers[37] and compensating missing parameters with GAFF.[38,39] The atomic charges for missing AmNA monomers were determined by the following method. First, a model molecule was prepared, in which two methyl-capped phosphate groups were added to the 5′- and 3′-ends of a nucleotide, respectively, and energetically optimized on MOE. Second, the AM1-BCC method was performed using *antechamber* from the AmberTools20 package.[40,41] Third, the atomic charges of the main chain atoms were manually modified to match those of the RNA monomers. The atom types and charges of the AmNA monomers used in this study are summarized in Table S3, and the atom names of the bridged part are shown in Figure S7.

A cut-off of 12Å was used for non-bonded interactions, while the particle mesh Ewald method[42] was used for electrostatic interactions.

Except for the heavy atoms in the nucleotides, each system underwent energetic minimization through 100 steps of steepest descent. Subsequently, the entire system was minimized using 5,000 steps of steepest descent. The SHAKE algorithm was implemented to restrain hydrogen bonds. All systems experienced gradual heating from 1 K to 300 K over 90 ps at a rate of 2 fs/step, using Langevin MD. The temperature increment was set at 1° every 150 steps. Upon reaching 300 K, each system was equilibrated for 100 ps at 2.5 fs/step using the RESPA algorithm. This was followed by 300 ps of MD, with structure sampling conducted every 100 ps to serve as the initial structures for subsequent product runs. Five runs were conducted for each polymer, each starting with a different initial structure. The process commenced at 300 K, with the system being heated to 500 K at an increase of 1 K every 200 ps at 2 fs/step using the LEAP algorithm; trajectories were recorded every 40 ps. GENESIS software was used for both energy minimization and MD simulations.[43,44]

BPS

A BPS is defined by the fulfillment of three criteria: (1) The mean distance between the hydrogen bond donor and acceptor atoms within the pair (N1 and N6 for adenine; N3 and O4 for thymine or uracil; N2, N1, and O6 for guanine; O2, N3, and N4 for cytosine)—must be less than 4.0 Å. (2) The angle between the two planes, each formed by the six atoms (N1, C2, N3, C4, C5, and C6) of the base rings of the pair, should exceed 170°. (3) The separation between the base pairs, as determined by CURVES+[45] analysis, must be greater than −1. These criteria were applied to MD simulation trajectories, wherein a step in the trajectory was substituted with the temperature set for the simulation.

DATA AND CODE AVAILABILITY

All nucleic acid sequences and T_m values collected for this study, as well as atom types and atomic charges for the MD simulations, are available in Excel files as supplemental information. Other data or codes are available from the first author (m-kuroda@nibiohn.go.jp) on reasonable request.

SUPPLEMENTAL INFORMATION

Supplemental information can be found online at https://doi.org/10.1016/j.omtn.2024.102272.

ACKNOWLEDGMENTS

We thank Suyong Re for his instructions and valuable suggestions on the MD package, GENESIS, and MD simulation. We would also like to thank Editage (www.editage.jp) for English language editing.
This research was supported by the Japan Agency for Medical Research and Development (AMED) Grant Numbers JP19am0401003, JP22gm1610010, and JP223fa727001, and the Japan Society for the Promotion of Science KAKENHI, Grant Number 21K14743.

AUTHOR CONTRIBUTIONS

M.K. built prediction models, performed MD simulations, analyzed the trajectories, and wrote the manuscript. Y.K. conceived the study, prepared the samples, and wrote the manuscript. M.H. performed T_m measurements by DSC. H.Y. prepared the samples. M.O. advised on the DSC experiments and results. C.N. advised on the model construction and edited the manuscript. K.M. supervised the study and edited the manuscript. All authors approved the final version of the manuscript.

DECLARATION OF INTERESTS

The authors declare no competing interests.

REFERENCES

1. Morihiro, K., Kasahara, Y., and Obika, S. (2017). Biological applications of xeno nucleic acids. Mol. Biosyst. *13*, 235–245.
2. Al Shaer, D., Al Musaimi, O., Albericio, F., and de la Torre, B.G. (2024). 2023 FDA TIDES (Peptides and Oligonucleotides) Harvest. Pharmaceuticals *17*, 243.
3. Baylot, V., Le, T.K., Taïeb, D., Rocchi, P., and Colleaux, L. (2024). Between hope and reality: treatment of genetic diseases through nucleic acid-based drugs. Commun. Biol. *7*, 489.
4. Nielsen, J.T., Stein, P.C., and Petersen, M. (2003). NMR structure of an α-L-LNA:RNA hybrid: structural implications for RNase H recognition. Nucleic Acids Res. *31*, 5858–5867.
5. Kauppinen, S., Vester, B., and Wengel, J. (2005). Locked nucleic acid (LNA): High affinity targeting of RNA for diagnostics and therapeutics. Drug Discov. Today Technol. *2*, 287–290.
6. Kaur, H., Babu, B.R., and Maiti, S. (2007). Perspectives on chemistry and therapeutic applications of Locked Nucleic Acid (LNA). Chem. Rev. *107*, 4672–4697.
7. Veedu, R.N., and Wengel, J. (2010). Locked nucleic acids: promising nucleic acid analogs for therapeutic applications. Chem. Biodivers. *7*, 536–542.
8. Obika, S., Rahman, S.M.A., Fujisaka, A., Kawada, Y., Baba, T., and Imanishi, T. (2010). Bridged Nucleic Acids: Development, Synthesis and Properties. Heterocycles *81*, 1347–1392.
9. Yahara, A., Shrestha, A.R., Yamamoto, T., Hari, Y., Osawa, T., Yamaguchi, M., Nishida, M., Kodama, T., and Obika, S. (2012). Amido-bridged nucleic acids (AmNAs): synthesis, duplex stability, nuclease resistance, and *in vitro* antisense potency. Chembiochem *13*, 2513–2516.
10. Yamamoto, T., Yahara, A., Waki, R., Yasuhara, H., Wada, F., Harada-Shibab, M., and Obika, S. (2015). Amido-bridged nucleic acids with small hydrophobic residues enhance hepatic tropism of antisense oligonucleotides *in vivo*. Org. Biomol. Chem. *13*, 3757–3765.
11. Setoguchi, K., Cui, L., Hachisuka, N., Obchoei, S., Shinkai, K., Hyodo, F., Kato, K., Wada, F., Yamamoto, T., Harada-Shiba, M., et al. (2017). Antisense Oligonucleotides Targeting Y-Box Binding Protein-1 Inhibit Tumor Angiogenesis by Downregulating Bcl-xL-VEGFR2/-Tie Axes. Mol. Ther. Nucleic Acids *9*, 170–181.
12. Uehara, T., Choong, C.J., Nakamori, M., Hayakawa, H., Nishiyama, K., Kasahara, Y., Baba, K., Nagata, T., Yokota, T., Tsuda, H., et al. (2019). Amido-bridged nucleic acid (AmNA)-modified antisense oligonucleotides targeting α-synuclein as a novel therapy for Parkinson's disease. Sci. Rep. *9*, 7567.
13. Shimojo, M., Kasahara, Y., Inoue, M., Tsunoda, S., Shudo, Y., Kurata, T., and Obika, S. (2019). A gapmer antisense oligonucleotide targeting SRRM4 is a novel therapeutic medicine for lung cancer. Sci. Rep. *9*, 7618.
14. Kanda, M., Kasahara, Y., Shimizu, D., Miwa, T., Umeda, S., Sawaki, K., Nakamura, S., Kodera, Y., and Obika, S. (2020). Amido-bridged Nucleic Acid-modified Antisense Oligonucleotides Targeting SYT13 to Treat Peritoneal Metastasis of Gastric Cancer. Mol. Ther. Nucleic Acids *22*, 791–802.
15. SantaLucia, J., Allawi, H.T., and Seneviratne, P.A. (1996). Improved Nearest-Neighbor Parameters for Predicting DNA Duplex Stability. Biochemistry *35*, 3555–3562.
16. Freier, S.M., Kierzek, R., Jaeger, J.A., Sugimoto, N., Caruthers, M.H., Neilson, T., and Turner, D.H. (1986). Improved free-energy parameters for predictions of RNA duplex stability. Proc. Natl. Acad. Sci. USA *83*, 9373–9377.

17. Sugimoto, N., Nakano, S., Katoh, M., Matsumura, A., Nakamuta, H., Ohmichi, T., Yoneyama, M., and Sasaki, M. (1995). Thermodynamic Parameters To Predict Stability of RNA/DNA Hybrid Duplexes. Biochemistry *34*, 11211–11216.

18. QIAGEN. Tm Prediction. https://geneglobe.qiagen.com/jp/tools/tm-prediction.

19. Cock, P.J.A., Antao, T., Chang, J.T., Chapman, B.A., Cox, C.J., Dalke, A., Friedberg, I., Hamelryck, T., Kauff, F., Wilczynski, B., and de Hoon, M.J.L. (2009). Biopython: freely available Python tools for computational molecular biology and bioinformatics. Bioinformatics *25*, 1422–1423.

20. McTigue, P.M., Peterson, R.J., and Kahn, J.D. (2004). Sequence-Dependent Thermodynamic Parameters for Locked Nucleic Acid (LNA)−DNA Duplex Formation. Biochemistry *43*, 5388–5405.

21. Markova, N., Cairns, S., Jankevics-Jones, H., Kaszuba, M., Caputo, F., and Parot, J. (2021). Biophysical Characterization of Viral and Lipid-Based Vectors for Vaccines and Therapeutics with Light Scattering and Calorimetric Techniques. Vaccines *10*, 49.

22. Wong, K.Y., and Pettitt, B.M. (2008). The pathway of oligomeric DNA melting investigated by molecular dynamics simulations. Biophys. J. *95*, 5618–5626.

23. Pande, V., and Nilsson, L. (2008). Insights into structure, dynamics and hydration of locked nucleic acid (LNA) strand-based duplexes from molecular dynamics simulations. Nucleic Acids Res. *36*, 1508–1516.

24. Lomzov, A.A., Vorobjev, Y.N., and Pyshnyi, D.V. (2015). Evaluation of the Gibbs Free Energy Changes and Melting Temperatures of DNA/DNA Duplexes Using Hybridization Enthalpy Calculated by Molecular Dynamics Simulation. J. Phys. Chem. B *119*, 15221–15234.

25. Hagedorn, P.H., Hansen, B.R., Koch, T., and Lindow, M. (2017). Managing the sequence-specificity of antisense oligonucleotides in drug discovery. Nucleic Acids Res. *45*, 2262–2282.

26. Dieckmann, A., Hagedorn, P.H., Burki, Y., Brügmann, C., Berrera, M., Ebeling, M., Singer, T., and Schuler, F. (2018). A Sensitive In Vitro Approach to Assess the Hybridization-Dependent Toxic Potential of High Affinity Gapmer Oligonucleotides. Mol. Ther. Nucleic Acids *10*, 45–54.

27. Wada, F., Yamamoto, T., Kobayashi, T., Tachibana, K., Ito, K.R., Hamasaki, M., Kayaba, Y., Terada, C., Yamayoshi, A., Obika, S., and Harada-Shiba, M. (2021). Drug discovery and development scheme for liver-targeting bridged nucleic acid antisense oligonucleotides. Mol. Ther. Nucleic Acids *26*, 957–969.

28. Ke, G., Meng, Q., Finley, T., Wang, T., Chen, W., Ma, W., Ye, Q., and Liu, T.Y. (2017). LightGBM: A Highly Efficient Gradient Boosting Decision Tree. Adv. Neural Inf. Process. Syst. *30*, 3149–3157.

29. Real, E., Liang, C., So, D., and Le, Q. (2020). AutoML-Zero: evolving machine learning algorithms from scratch. In Proceedings of the 37th International Conference on Machine Learning (PMLR), pp. 8007–8019.

30. Mulpuru, V., and Mishra, N. (2021). In silico prediction of fraction unbound in human plasma from chemical fingerprint using automated machine learning. ACS Omega *6*, 6791–6797.

31. Wang, C., Wu, Q., Weimer, M., and Zhu, E. (2021). FLAML: A Fast and Lightweight AutoML Library. Proc. Mach. Learn. Syst. *3*, 434–447.

32. Dabin, A., and Stirnemann, G. (2023). Toward a molecular mechanism of complementary RNA duplexes denaturation. J. Phys. Chem. B *127*, 6015–6028.

33. Sanstead, P.J., Stevenson, P., and Tokmakoff, A. (2016). Sequence-Dependent Mechanism of DNA Oligonucleotide Dehybridization Resolved through Infrared Spectroscopy. J. Am. Chem. Soc. *138*, 11792–11801.

34. Settles, B. (1994), Computer Sciences Technical Report 1648. Active Learning Literature Survey, *15* (University of Wisconsin-Madison), pp. 1–67. http://digital.library.wisc.edu/1793/60660.

35. Akiba, T., Sano, S., Yanase, T., Ohta, T., and Koyama, M. (2019). Optuna: a next-generation hyperparameter optimization framework. In Proceedings of the 25th ACM SIGKDD International Conference on Knowledge Discovery & Data Mining, pp. 2623–2631.

36. (2022). Molecular Operating Environment (MOE) (Chemical Computing Group ULC).

37. Cornell, W.D., Cieplak, P., Bayly, C.I., Gould, I.R., Merz, K.M., Ferguson, D.M., Spellmeyer, D.C., Fox, T., Caldwell, J.W., and Kollman, P.A. (1995). A Second Generation Force Field for the Simulation of Proteins, Nucleic Acids, and Organic Molecules. J. Am. Chem. Soc. *117*, 5179–5197.

38. Wang, J., Wolf, R.M., Caldwell, J.W., Kollman, P.A., and Case, D.A. (2004). Development and testing of a general amber force field. J. Comput. Chem. *25*, 1157–1174.

39. Case, D.A., Belfon, K., Ben-Shalom, I.Y., Brozell, S.R., Cerutti, D.S., Cheatham, T.E., III, Cruzeiro, V.W.D., Darden, T.A., Duke, R.E., Giambasu, G., et al. (2020). AMBER 2020 (University of California).

40. Jakalian, A., Bush, B.L., Jack, D.B., and Bayly, C.I. (2000). Fast, efficient generation of high-quality atomic charges. AM1-BCC model: I. Method. J. Comput. Chem. *21*, 132–146.

41. Jakalian, A., Jack, D.B., and Bayly, C.I. (2002). Fast, efficient generation of high-quality atomic charges. AM1-BCC model: II. Parameterization and Validation. J. Comput. Chem. *23*, 1623–1641.

42. Darden, T., York, D., and Pedersen, L. (1993). Particle mesh Ewald: An N•log(N) method for Ewald sums in large systems. J. Chem. Phys. *98*, 10089–10092.

43. Kobayashi, C., Jung, J., Matsunaga, Y., Mori, T., Ando, T., Tamura, K., Kamiya, M., and Sugita, Y. (2017). GENESIS 1.1: A hybrid-parallel molecular dynamics simulator with enhanced sampling algorithms on multiple computational platforms. J. Comput. Chem. *38*, 2193–2206.

44. Jung, J., Mori, T., Kobayashi, C., Matsunaga, Y., Yoda, T., Feig, M., and Sugita, Y. (2015). GENESIS: A hybrid-parallel and multi-scale molecular dynamics simulator with enhanced sampling algorithms for biomolecular and cellular simulations. Wiley Interdiscip. Rev. Comput. Mol. Sci. *5*, 310–323.

45. Blanchet, C., Pasi, M., Zakrzewska, K., and Lavery, R. (2011). CURVES+ web server for analyzing and visualizing the helical, backbone and groove parameters of nucleic acid structures. Nucleic Acids Res. *39*, W68–W73.

OMTN, Volume *35*

Supplemental information

Construction of a T_m-value prediction model and molecular dynamics study of AmNA-containing gapmer antisense oligonucleotide

Masataka Kuroda, Yuuya Kasahara, Masako Hirose, Harumi Yamaguma, Masayuki Oda, Chioko Nagao, and Kenji Mizuguchi

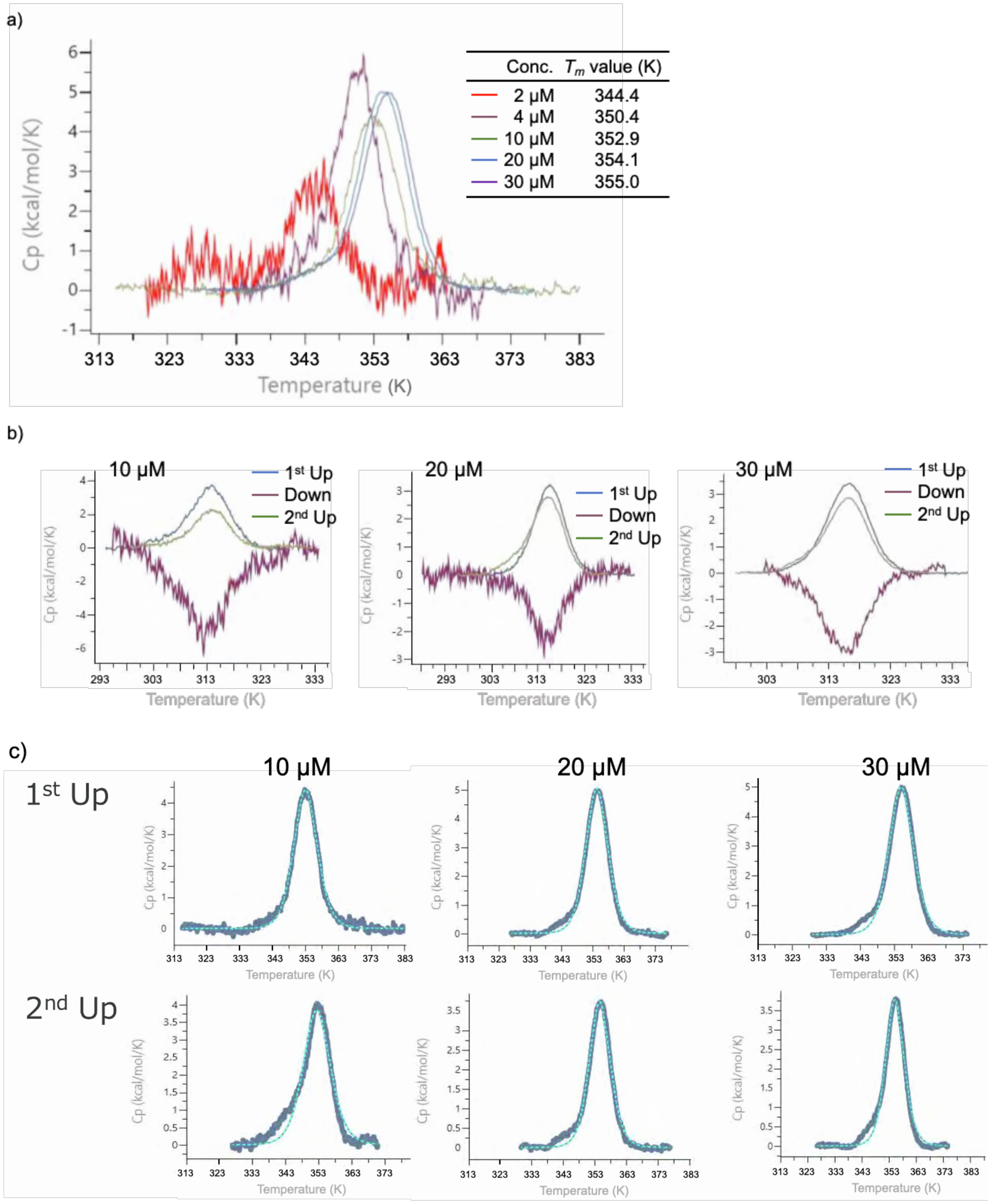

Figure S1. Differential scanning calorimetry (DSC) charts obtained from one sample.

(a) The analyzed lines of five different sample concentrations are superimposed. (b) The analysis lines for two temperature-increase and one temperature-decrease measurements are superimposed to check reversibility for three different sample concentrations. (c) The lines for two temperature-increase

measurements are superimposed to check symmetry for three different sample concentrations. The solid and broken lines indicate the measurements and the fitted line by a function, respectively.

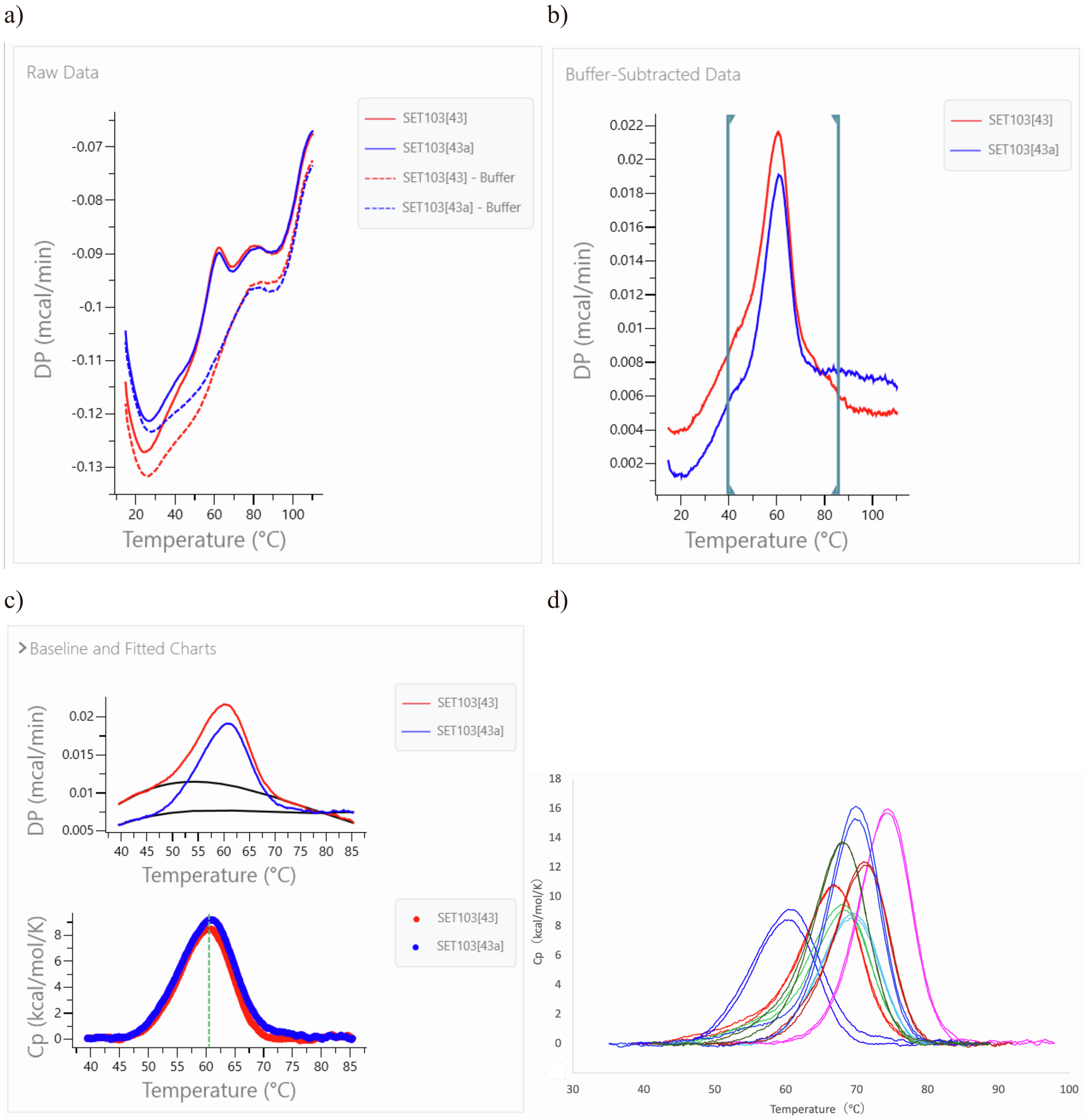

Figure S2. Measurement and Analysis Data by DSC

(a) Raw data collected for one sample. Two measurements and buffer scan data are superimposed. (b) measurement data from which the buffer data is subtracted. The area between the green bars is used for analysis. (c) Analyzed DSC data. (d) Analyzed data for eight samples are superimposed.

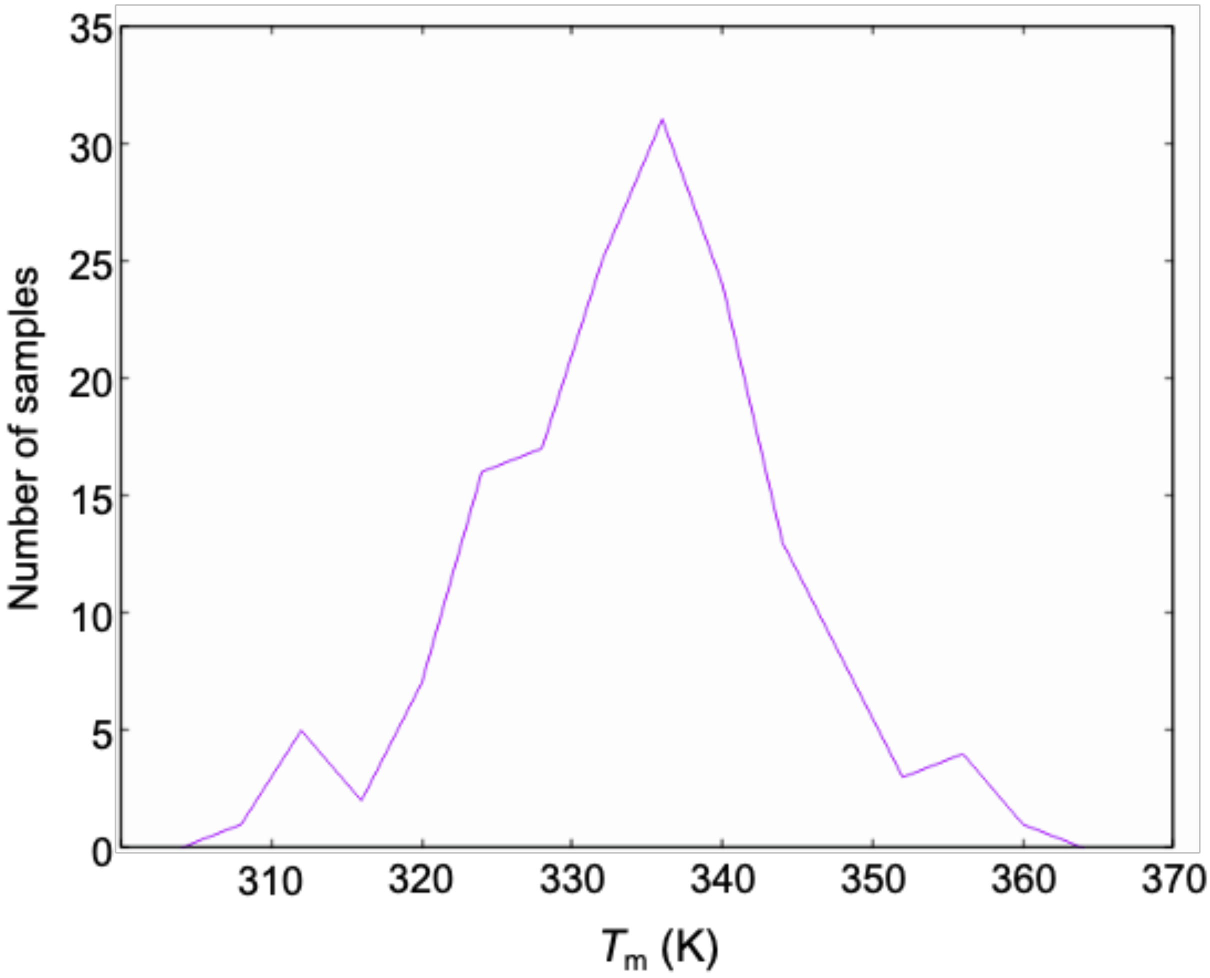

Figure S3. The Distribution of the Obtained T_m Values

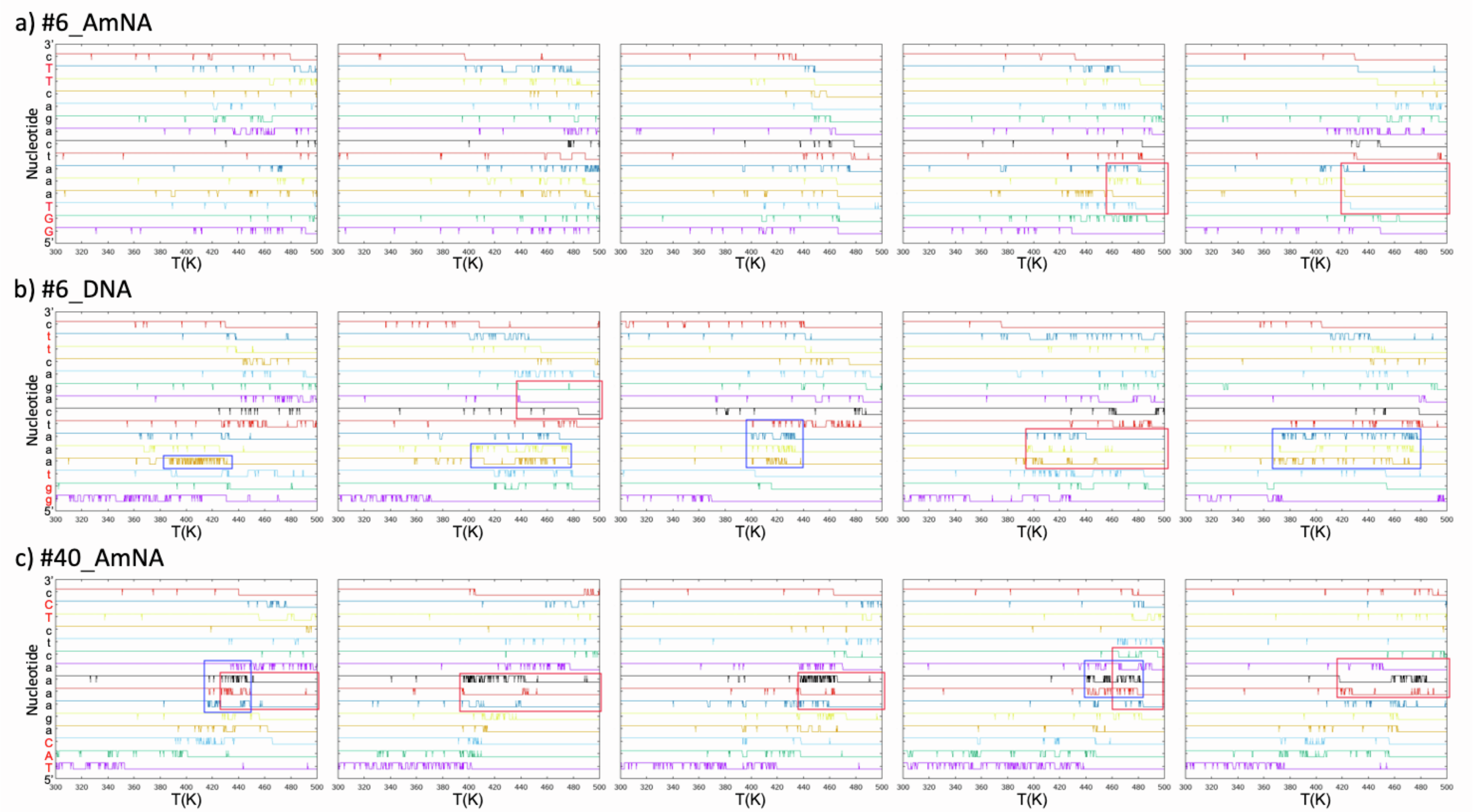

a) #6_AmNA
b) #6_DNA
c) #40_AmNA
Nucleotide
T(K)

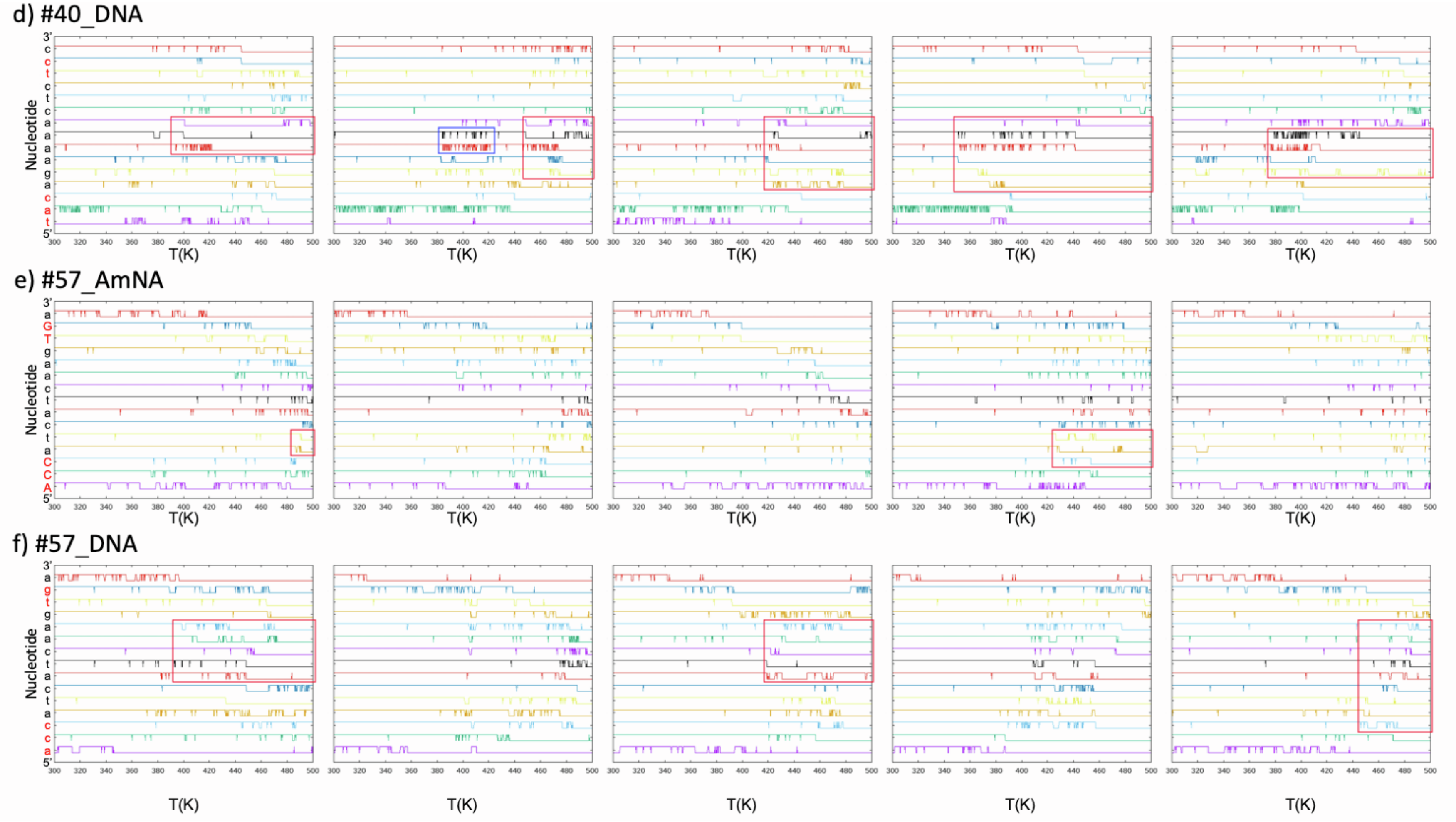

Figure S4. BPS Formation/Destruction Obtained from All MD Runs

(a) #6_AmNA, (b) #6_DNA, (c) #40_AmNA, (d) #40_DNA, (e) #57_AmNA, (f) #57_DNA. The results of run numbers one to five from the left side are displayed. The vertical axis represents the nucleotide from the 5' to the 3' end, with capital and small letters representing AmNA and DNA. The letter in red is the mutated position. The horizontal axis is the temperature (K) set by the MD program in the run. The top and bottom positions

of each line indicate BPS formation and non-formation, respectively. The red rectangle shows the duplex break in the middle of the oligomer. The blue rectangle shows the fray that occurs in the middle of the duplex.

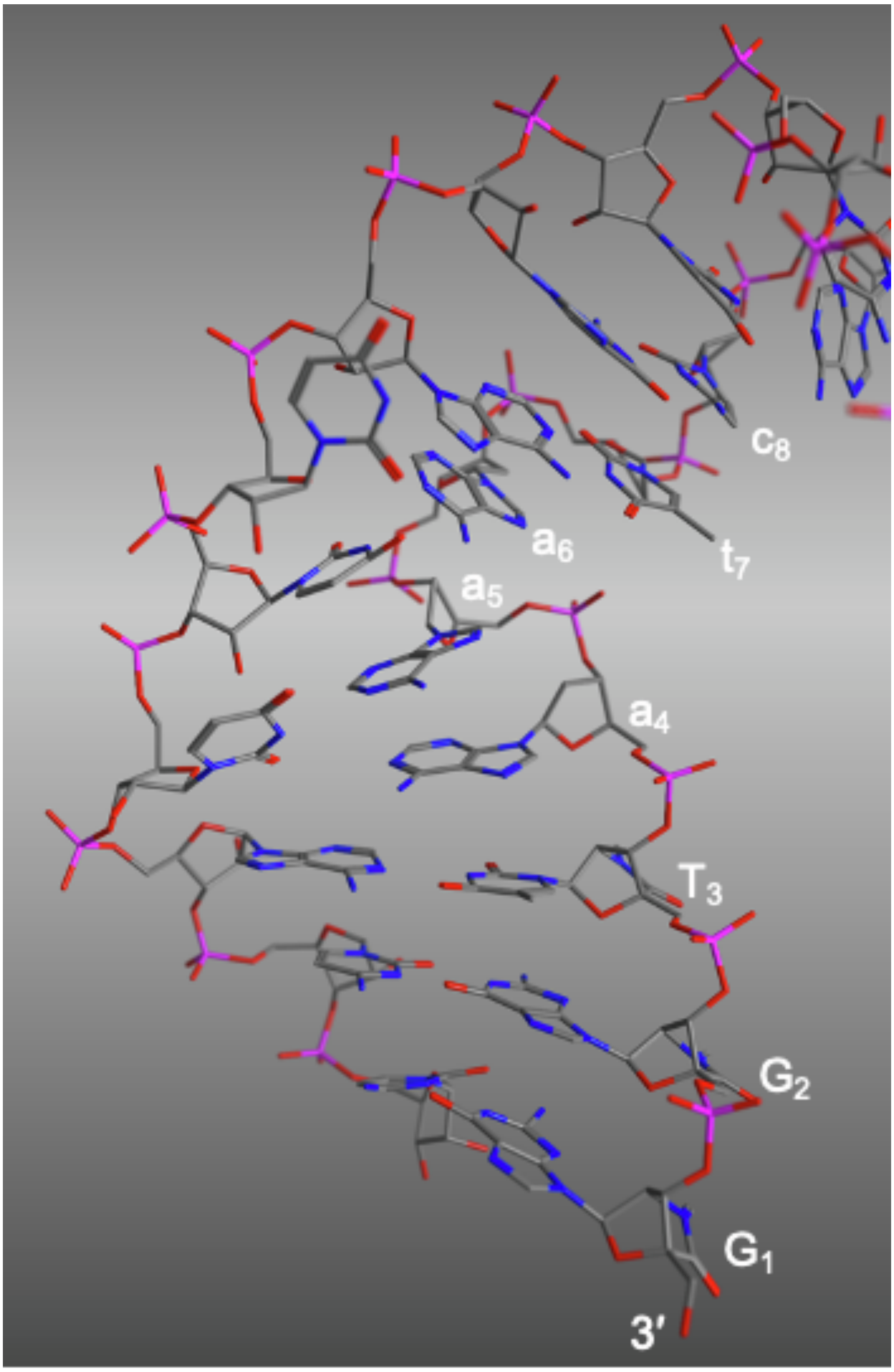

Figure S5. A snapshot of the MD trajectory for #6_AmNA 5 with the simulation temperature set to 420K, corresponding to the left side of the red rectangle in Figure 3A.

The base pair steps of the three nucleotides, a_4-a_6, are dissociated.

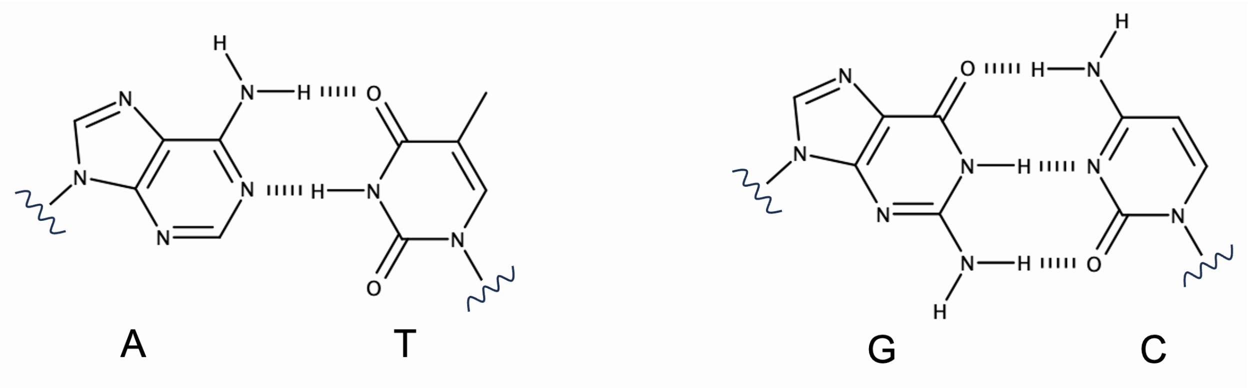

Figure S6. Interaction Modes of AT and GC Pairs

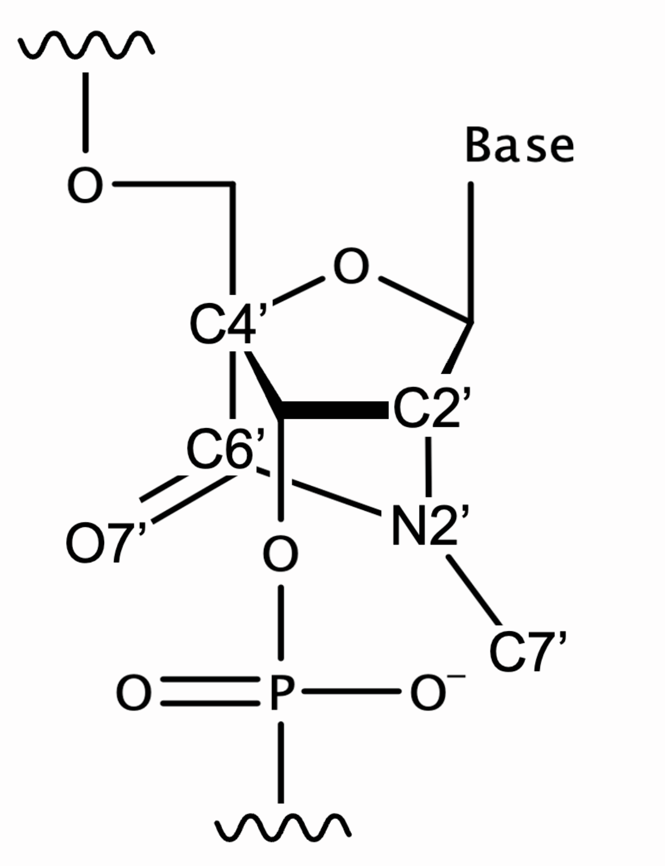

Figure S7. Atom Names of the Bridged Part in AmNA Monomer Used in Topology File for Molecular Dynamics

Table S1. T_m values for all oligonucleotides observed by DSC

Table S2. Feature importance analysis

[a]The importance values were extracted from ten models created for the performance test. They are presented as a percentage of the total importance.

Table S3. Atomic types and charges of AmNA monomers used in molecular dynamics

Atom names for the bridged part are shown in Figure S7.
